# Chimeric Antigen Receptor (CAR) T-Cell Therapy in Hematologic Malignancies: Clinical Implications and Limitations

**DOI:** 10.3390/cancers16081599

**Published:** 2024-04-22

**Authors:** Philipp Blüm, Sabine Kayser

**Affiliations:** 1 Institute of Transfusion Medicine and Immunology, Medical Faculty Mannheim, Heidelberg University, German Red Cross Blood Service Baden-Württemberg-Hessen, 68167 Mannheim, Germany; philipp.bluem@medma.uni-heidelberg.de; 2 NCT Trial Center, National Center of Tumor Diseases, Heidelberg University Hospital and German Cancer Research Center (DKFZ), 69120 Heidelberg, Germany

**Keywords:** chimeric antigen receptor T-cells, CAR T-cells, tisagenlecleucel, axicabtagene ciloleucel, brexucabtagene autoleucel, idecabtagene vicleucel, lisocabtagene maraleucel, ciltacabtagene autoleucel, cytokine release syndrome, immune effector cell-associated neurotoxicity syndrome

## Abstract

**Simple Summary:**

This is a comprehensive overview of the approved chimeric antigen receptor T-cell products (CAR T-cells), their specific indications, treatment-related side effects, and scientific challenges. CAR T-cells have been introduced to modern hematology over the last years and have changed treatment outcomes in heavily pretreated patients with B-cell and plasma cell malignancies. To date, six commercially available CAR T-cell products have been approved by the U.S. Food and Drug Administration (FDA) and by the European Medicines Agency (EMA). These advanced therapeutic medicinal products induce strong treatment effects but can also cause adverse events that can potentially be life-threatening. Therefore, a thorough understanding of CAR T-cell function and characteristics is essential for safety and efficacy. This review provides a comprehensive overview of the clinical applications of CAR T-cells, focusing on the approved products and emphasizing their benefits but also indicating limitations and challenges.

**Abstract:**

Chimeric antigen receptor (CAR) T-cell therapy has become a powerful treatment option in B-cell and plasma cell malignancies, and many patients have benefited from its use. To date, six CAR T-cell products have been approved by the FDA and EMA, and many more are being developed and investigated in clinical trials. The whole field of adoptive cell transfer has experienced an unbelievable development process, and we are now at the edge of a new era of immune therapies that will have its impact beyond hematologic malignancies. Areas of interest are, e.g., solid oncology, autoimmune diseases, infectious diseases, and others. Although much has been achieved so far, there is still a huge effort needed to overcome significant challenges and difficulties. We are witnessing a rapid expansion of knowledge, induced by new biomedical technologies and CAR designs. The era of CAR T-cell therapy has just begun, and new products will widen the therapeutic landscape in the future. This review provides a comprehensive overview of the clinical applications of CAR T-cells, focusing on the approved products and emphasizing their benefits but also indicating limitations and challenges.

## 1. Introduction

Immunotherapies are an integral part of modern medicine and have revolutionized therapeutic options in many different areas [1,2,3,4]. In a wider sense, immunotherapy comprises every medical attempt to influence the patient’s immune system in a favorable manner [1]. This includes immunosuppressive measures (e.g., glucocorticoids, chemotherapies) [4,5], as well as attempts to modulate (e.g., immunomodulatory drugs “IMiD”) and activate the immune system. Monoclonal antibodies (mAbs) [1,3], antibody–drug conjugates (ADCs) [6], and bi-specific T-cell engagers (BiTEs) [2,7] use the specific binding abilities of the antibody system to target a defined cell of interest and to induce an antigen-dependent treatment effect. In addition, allogeneic hematopoietic stem cell transplantation (HSCT) and donor lymphocyte infusion (DLI) [8,9,10,11] represent early forms of cell therapies, in which a complete immune system or immune cells of a donor are transplanted into a recipient, respectively. Over the last years, a new subtype of immune therapies has found its way into clinical routine: immune effector cell (IEC) therapies. IECs are characterized by a collection step of immune cells and a manufacturing step for the production of the final therapeutic product [1,3].

In this review, we will focus on chimeric antigen receptor (CAR) T-cells, which represent a specific type of IECs, which have been introduced in hematology/oncology over the last years [12,13,14,15,16,17,18,19,20,21,22,23]. The term “chimeric” refers to the ancient Greek mythologic creature “chimera”, a hybrid animal composed of different species. In this regard, “chimeric receptors” are genetically engineered hybrids of antibody-derived variable regions and T-cell receptor (TCR)-derived signaling domains.

We provide a concise overview of approved CAR T-cell products and discuss advantages and disadvantages as well as relevant side effects. Finally, we discuss limitations and obstacles that have to be solved in the near future.

## 2. A Short History of Immune Effector Cell Therapy

The idea of immune effector cell therapy, also known as “adoptive cell transfer”, and its potential anti-tumor effect had been already recognized in basic research projects starting in the 1950s [24,25,26,27]. The immune system came increasingly into focus in cancer research due to the “graft-versus-leukemia” effect (GVL) [10,28], anti-tumor effects of tumor-infiltrating lymphocytes (TILs) [27,29,30], and DLI [8,9]. This was supported by a better understanding of the function and composition of the T-cell immune system on one hand and by improved gene transfer techniques on the other hand [31].

Current gene therapy facilitates the genetic reprogramming of T-cells, which can be used to optimize tumor antigen recognition, to improve cell survival and expansion, and to overcome T-cell death, anergy, and immune suppression [31]. These goals can be achieved by designing artificial antigen receptors, which can represent physiological major histocompatibility complex (MHC)-restricted T-cell receptors (TCRs) or non-MHC-restricted CARs [31,32].

## 3. Chimeric Antigen Receptor T-Cell Development

The history of CAR T-cells dates back more than three decades to the end of the 1980s (Table 1).

Several research groups worked independently on the functional expression of CARs. Kuwana et al. first described a CAR composed of immunoglobulin-derived variable regions and TCR-derived constant regions in 1987 [33]. Variants of CAR T-cell constructs were also published by Becker et al. [34], Eshhar et al. [35,36], and Goverman et al. [37] in the following years.

In 1993, Eshhar et al. described the construction of a later known “first-generation” CAR T-cell product and already outlined its potential use in medicine [38]. In contrast to the original antibody-based double-chain chimeric receptors, this newly generated receptor type only consisted of a single-chain variable fragment (scFv) of a given antibody, which was linked with gamma or zeta chains of the TCR complex. The huge advantage of these chimeric receptors lies in the ability to directly bind their corresponding antigen on the cell surface independently of antigen processing or MHC-restricted presentation [32]. Additionally, CAR T-cell receptors are able to recognize protein, carbohydrate, and glycolipid antigens, whereas TCR T-cells mostly target protein antigens [58,59,60]. Furthermore, mouse CAR T-cells were able to provide an acceptable anti-tumor effect in vivo for different tumor antigens (e.g., ERBB2, CEA, TAG-72, FBP) [61,62,63,64,65,66].

Although these receptor constructs can specifically bind their corresponding target antigen, they are not able to fully activate naïve unprimed T-cells [32]. The results of the first CAR T-cell clinical trials in patients with metastatic solid tumors (such as ovarian cancer, neuroblastoma, renal cell carcinoma, etc.) were published in 2006. However, they showed no relevant anti-tumor effects and a poor persistency of CAR T-cells in patients [39,40,41]. This was in part due to the lack of costimulatory domains. Especially naïve CD4+ T-cells depend on costimulation (e.g., via CD28) to undergo full activation in response to antigen presentation and to escape anergy or apoptosis [67,68,69,70,71].

**Figure 1:**
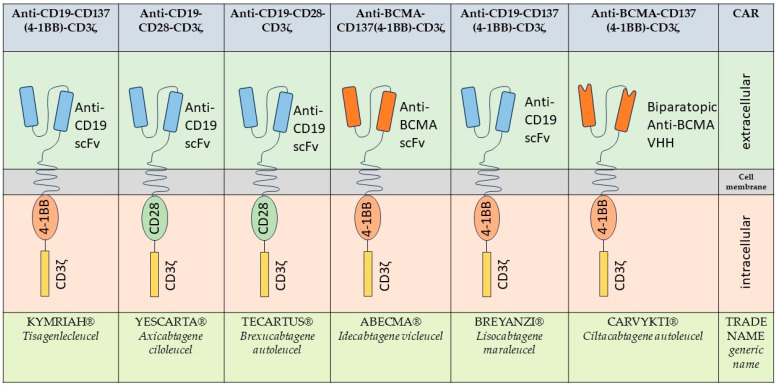
Overview of the approved CAR T-cell constructs [12,13,14,15,16,17,18,19,20,21,22,23,80].

To overcome these issues, the “second-generation” CAR T-cell products were introduced in which a costimulatory domain (e.g., CD28, 4-1BB) was fused to the receptor construct [42,43,58,72,73,74,75,76,77]. With the support of these costimulatory domains, CAR T-cells were now able to meet all biological requirements for T-cell priming and amplification, which resulted in an effective immune response and represents a central aspect of the development of immune effector cell therapy [31,43,75,78]. Five out of the six approved and commercially available CAR T-cell products are all members of this “second generation” (Figure 1) [76,79]. The sixth CAR T-cell product consists of a CD3ζ signaling domain and a 4-1BB costimulatory domain. In contrast to the other CAR T-cell constructs, the antigen binding domain consists of two nanobody heavy chains (VHH) [79].

By adding additional costimulatory domains to the receptor construct, “third-generation” CAR T-cell products are under development but have not yet found their way to clinics [76,79].

Besides this “classical” CAR T-cell design, researchers have developed advanced CAR platforms to improve safety and efficacy, including multispecific CARs, combinatorial antigen sensing CARs, drug-regulated and adapter CARs, and many more [76,79]. These approaches will broaden the impact of future CAR T-cell therapies.

## 4. Approved CAR T-Cell Products

As of February 2024, six commercially available CAR T-cell products have been approved by the U.S. Food and Drug Administration (FDA) and the European Medicines Agency (EMA; Figure 1).

The corresponding indications of these products are summarized in Table 2.

Table 3 gives an overview of the underlying clinical trials with their most relevant results.

### 4.1. Tisagenlecleucel (Kymriah^®^)

In 2009, Milone et al. described the engineering of an anti-CD19-CD137(4–1BB)-CD3ζ CAR T-cell product, which exhibited a strong antileukemic effect and prolonged (>6 months) survival in vivo [74]. In 2011, the first in-patient results of this CAR T-cell product (CTL019) were published showing a strong and long-lasting antileukemia effect after infusion of CTL019 CAR T-cells [116,117].

In 2013, investigators reported the primary results of the application of CTL019 CAR T-cells in two children with relapsed and refractory pre-B-cell acute lymphoblastic leukemia (ALL) [118,119]. After infusion of 1.4 × 10^6^ to 1.2 × 10^7^ CTL019 cells per kilogram of body weight, both patients responded well and initially achieved a complete remission (CR) of the ALL [118]. Both patients developed cytokine release syndrome (CRS) and B-cell aplasia, with one child experiencing severe CRS, which had to be treated with etanercept and tocilizumab on an intensive care unit [118]. Although both children responded initially very well to CTL019 therapy, one child developed a CD19- (negative) relapse two months after infusion [118]. The final results were published in 2014 [120]. A total of thirty children and adults (twenty-five children (5–22 years of age); five adults (26–60 years of age)) with relapsed and refractory CD19+ ALL were treated with CTL019. Sustained remission was achieved with a 6-month event-free survival (EFS) rate of 67% and an overall survival (OS) rate of 78%. All patients developed CRS, while 27% experienced severe CRS, which was associated with a higher disease burden prior to infusion [120].

Based on these remarkable results, a single-cohort, multicenter study of tisagenlecleucel (formerly known as CTL019) was conducted [44,121,122]. Between April 2015 and July 2019, 97 patients with relapsed or refractory B-cell ALL were enrolled, and 79 (81%) received tisagenlecleucel (median age of 11 years (range, 3 to 24); the patients underwent a median of three previous therapies (range, 1 to 8)) [121,123]. The overall response rate (ORR) within 3 months was 82% (95% CI, 72% to 90%) [123]. In patients who achieved a CR with incomplete hematologic recovery (CRi), the median duration of remission (DOR) was not reached [123]. Estimated relapse-free survival (RFS) was 58% (95% CI, 43% to 70%) at 24 months and 52% at 36 months (95% CI, 37% to 66%) [124]. The median event-free survival (EFS) of all infused patients was 24 months (95% CI, 9.2 months to not reached), and the median EFS among responders was not reached (95% CI, 18.7 months to not reached) [123].

In August 2017, based on preliminary results [121], the FDA approved tisagenlecleucel for the treatment of patients up to 25 years of age with B-cell precursor ALL that is refractory in second or later relapse [125]. In addition, tisagenlecleucel was approved by the EMA in August 2018 [23].

In April 2018, the FDA extended the approval by adding the treatment of adult patients with relapsed or refractory (r/r) large B-cell lymphoma (DLBCL) after two or more lines of systemic therapy as a new indication [17]. This extension was justified by a phase 2, single-arm, multicenter trial in adult patients with DLBCL [45]. In this study, 93 adult patients with r/r DLBCL were enrolled who were ineligible for or had disease progression after high-dose chemotherapy and autologous hematopoietic stem cell transplantation [52]. The ORR was 52% (95% CI, 41% to 62%), with CR in 40% and partial responses (PRs) in 12%. The 12-month RFS was 65% (79% among patients with CR). Common side effects, such as CRS and immune effector cell-associated neurotoxicity syndrome (ICANS), were similar as compared to patients with ALL [52]. This indication was already included in the EMA’s marketing authorization of August 2018 [23].

To investigate the effectiveness of tisagenlecleucel in patients with r/r follicular lymphoma (FL), a phase 2, single-arm, multicenter, open-label trial was initiated in November 2018 [47]. For the interim analysis, 97 of 98 enrolled patients received tisagenlecleucel. The complete response rate (CRR) was 69.1% (95% CI, 58.8% to 78.3%), and the ORR was 86.2% (95% CI, 77.5% to 92.4%) [124]. The safety profile was comparable to other indications without treatment-related deaths [124,126].

On 27 May 2022, the FDA approved tisagenlecleucel for the treatment of adult patients with r/r FL after two or more lines of systemic therapy [17]. Accordingly, the EMA marketing authorization for the use of tisagenlecleucel was also extended [23].

### 4.2. Axicabtagene Ciloleucel (Yescarta^®^)

Axicabtagene ciloleucel (formerly known as KTE-C19) is a second-generation CAR T-cell construct consisting of an anti-CD19 scFv, derived from the FMC63 mouse hybridoma, a human CD28 costimulatory domain, and an intracellular TCR-CD3ζ signaling domain [127,128]. In a phase 1/2 clinical trial, Rosenberg et al. were able to show successful and long-lasting responses in patients with r/r B-cell non-Hodgkin lymphomas (NHLs) [127,129,130,131].

Axicabtagene ciloleucel was investigated in another phase 1/2 trial (NCT02348216—ZUMA-1) [53]. In the phase 1 part, nine patients were enrolled, and the results demonstrated sufficient safety and feasibility of production. Additionally, a robust CAR T-cell expansion and durable clinical responses could be observed, which led to the initiation of the pivotal ZUMA-1 phase 2 trial [132]. The results of phase 2 of ZUMA-1 and an updated analysis of phase 1 with one year of follow-up were published in 2017 [133]. Overall, the trial included 111 patients with either refractory DLBCL (cohort 1) and primary mediastinal B-cell lymphoma (PMBCL) or transformed FL (cohort 2). Refractory disease was defined as progressive or stable disease as the best response to the most recent chemotherapy or disease progression or relapse within 12 months after high-dose chemotherapy and autologous HSCT [133]. Axicabtagene ciloleucel was produced for 110 participants and administered to 101 [133]. After 6 months of follow-up, the ORR was 82% (95% CI, 72% to 89%) [133]. Among patients with response to therapy, the CRR was 52% [133]. The median OS was not reached (95% CI, 12.0 months to not reached), with OS rates of 78% (95% CI, 69% to 85%) at 6 months, 59% (95% CI, 49% to 68%) at 12 months, and 52% (95% CI, 41% to 62%) at 18 months [133,134]. Based on these results, the FDA approved axicabtagene ciloleucel for the treatment of adult patients with r/r large B-cell lymphoma after two or more lines of systemic therapy [12]. In June 2018, the EMA granted authorization for the EU with a similar indication [18].

Interestingly, follow-up analysis of the ZUMA-1 trial did not only show a potent and constringent early response to therapy but also a long-term disease-specific estimated survival of 51% at 5 years with a polyclonal B-cell recovery after therapy [134]. These findings raise hope for a potentially curative treatment effect in a subset of patients with large B-cell lymphomas [134].

In an international, randomized, phase 3 clinical trial, the efficacy of axicabtagene ciloleucel was compared with standard care (SOC—defined as two or three cycles of platinum-based chemotherapy, followed by high-dose chemotherapy and autologous HSCT) as a second-line treatment in patients with early r/r large B-cell lymphoma (NCT03391466—ZUMA-7) [57,82]. A total of 359 patients with large B-cell lymphomas that were refractory to or had relapsed within 12 months after first-line chemoimmunotherapy were enrolled and randomly assigned, in a 1:1 ratio, to receive axicabtagene ciloleucel or SOC [82]. In total, 180 patients were assigned to the axicabtagene ciloleucel group, 178 underwent leukapheresis, and 170 received the CAR T-cell product. In the SOC group, 168 (94%) patients received a platinum-based salvage chemotherapy, and 64 (36%) were treated with high-dose chemotherapy and autologous HSCT [82]. The median EFS was significantly longer in the axicabtagene ciloleucel group (8.3 months; 95% CI, 4.5 months to 15.8 months) as compared to that in the SOC group (2.0 months; 95% CI, 1.6 months to 2.8 months) [82]. The estimated EFS at 24 months was 41% (95% CI, 33% to 48%) in the CAR T-cell group vs. 16% (95% CI, 11% to 22%) in the SOC group [82]. At a median follow-up of 47.2 months, the OS was significantly higher after treatment with axicabtagene ciloleucel as compared to SOC [135]. The estimated 4-year OS rate was 54.6% (95% CI, 47.0% to 61.6%) with axicabtagene ciloleucel as compared to 46.0% (95% CI, 38.4% to 53.2%) with SOC [135]. The median OS was not reached in the axicabtagene ciloleucel group (95% CI, 28.6 months to not reached) and was 31.1 months in the SOC group (95% CI, 17.1 months to not reached) [135].

In summary, treatment with axicabtagene ciloleucel resulted in a significant (27.4%) reduction in the risk of death and an improvement in survival of 8.6% at 4 years [135].

To investigate the efficacy of axicabtagene ciloleucel in patients with r/r indolent NHL, a phase 2 multicenter study was conducted (NCT03105336—ZUMA-5) [54,136]. In this study, axicabtagene ciloleucel induced a high ORR of 92% (95% CI, 85–97%) and CR in 74% of the patients. Among patients with FL, 94% (95% CI, 87–97%) had an OR, of whom 79% achieved a CR [136]. Among patients with marginal zone lymphoma (MZL) 85% (95% CI, 62–97%) had an OR, of whom 55% experienced a CR [136]. Axicabtagene ciloleucel was able to induce long-lasting responses, with 59% of patients having an ongoing response at data cutoff (median follow-up of 23.3 months) [136]. Thus, the FDA and EMA amended the approval for axicabtagene ciloleucel, accordingly [12,18].

### 4.3. Brexucabtagene Autoleucel (Tecartus^®^)

Brexucabtagene autoleucel (formerly known as KTE-X19) is an anti-CD19 CAR T-cell product that expresses the same CAR T-cell receptor construct as axicabtagene ciloleucel (anti-CD19-CD28-CD3ζ) [88]. However, the manufacturing process of brexucabtagene autoleucel is different as compared to axicabtagene ciloleucel in a relevant step [88]. During the manufacturing process of brexucabtagene autoleucel, circulating CD19 expressing (malignant) cells are removed to reduce the possible activation and exhaustion of anti-CD19 CAR T-cells during the ex vivo manufacturing process [88]. Thus, brexucabtagene autoleucel was specifically designed for the treatment of leukemic CD19-positive malignant diseases, e.g., mantle cell lymphoma (MCL) and B-cell leukemias [88].

In a single-group, multicenter, phase 2 clinical trial, the efficacy of brexucabtagene autoleucel was evaluated in patients with r/r MCL (NCT02601313—ZUMA-2) [83,88]. Previous treatment consisted of up to five prior regimens, including anthracycline- or bendamustine-containing chemotherapy, an anti-CD20 mAb, and Bruton’s tyrosine kinase inhibitory therapy with ibrutinib or acalabrutinib [88]. From October 2016 to April 2019, a total of 74 patients were assigned to the ZUMA-2 clinical trial. The CAR T-cell product could be successfully manufactured for 71 patients (96%) and infused in 68 patients (92%) [88]. After a follow-up of at least 7 months, 93% of the patients (95% CI, 84% to 98%) had an objective response to therapy, with 67% (95% CI, 53% to 78%) showing a CR [88]. At 12 months, the estimated PFS and OS were 61% and 83%, respectively [88].

In addition, the efficacy and safety of brexucabtagene autoleucel was investigated in a phase 1 study in adult patients with r/r B-ALL [84]. Overall, 54 patients were included, and brexucabtagene autoleucel could be manufactured for all patients, with a median time from leukapheresis to delivery of 15 days [91]. In total, 45 of 54 (83%) patients were treated with brexucabtagene autoleucel. At a median follow-up of 22.1 months, the ORR was 69%, with 53% of patients achieving CR and 16% achieving CRi [89]. No dose limiting toxicities (DLTs) were observed, and the adverse events (AEs) were similar to those in previous studies of anti-CD19 CAR T-cell therapies [89].

In phase 2 of the ZUMA-3 study, 71 patients were enrolled. The CAR T-cell product was successfully manufactured for 65 (92%) and could be infused into 55 (77%) patients [92]. Thirty-nine patients (71%) achieved a CR or CRi [90]. Moreover, 42 (76%) of all treated patients became MRD-negative, with a high rate of MRD negativity among responders (*n* = 38/39; 97%) [90].

Based on these results, brexucabtagene autoleucel was approved by the FDA and EMA for the treatment of adult patients with r/r MCL and r/r B-ALL [13,19].

### 4.4. Idecabtagene Vicleucel (Abecma^®^)

Idecabtagene vicleucel (formerly known as bb2121) is an autologous CAR T-cell product, which was developed for the treatment of patients with r/r multiple myeloma (MM) [96,137,138]. In contrast to other approved CAR T-cell products that target the CD19 antigen, idecabtagene vicleucel contains an anti-BCMA single-chain variable fragment, targeting the B-cell maturation antigen (BCMA) [137,138,139]. The BCMA is highly expressed on MM cells, plasma cells, and mature B-cells, whereas CD19 is only expressed on a small fraction of myeloma cells and is therefore not a suitable target antigen [139,140,141].

Idecabtagene vicleucel is produced by the transduction of autologous T-cells with a second-generation CAR consisting of an anti-BCMA scFv, a 4-1BB costimulatory domain, and a CD3ζ signaling domain [138].

In a multicenter phase 1 study, 36 adult patients with r/r MM were enrolled [94,137]. The patients had to be refractory to at least three prior lines of therapy, including a proteasome inhibitor and an IMiD [137]. Overall, 33 of 36 (92%) patients received the manufactured CAR T-cell product. The ORR was 85% (95% CI, 68.1% to 94.9%), with 45% having a CR (9%) or stringent CR (36%). A strong expansion of CAR T-cells in vivo could be observed, and a durable persistence of CAR T-cells could be achieved, with 96%, 86%, 57%, and 20% of the patients having detectable CAR T-cells at 1, 3, 6, and 12 months, respectively [137]. The median response duration was 10.9 months [137]. Regarding the safety profile, CRS and ICANS frequencies seemed to be lower in comparison to anti-CD19-CAR T-cell therapies [137].

A single-group, phase 2 study enrolled adult patients with r/r MM, who had received at least three previous treatment lines (incl. IMiDs, proteasome inhibitors, anti-CD38-mAb) [91,96]. Overall, 140 patients were enrolled, of whom 128 received the final CAR T-cell product (NCT03361748—KarMMa) [91,96]. At a median follow-up of 13.3 months, 94 of 128 patients (73%) had a response, and 33% had a CR or stringent CR [96]. In addition, 52% of the patients achieved at least a very good partial response (VGPR) [96]. The estimated median of PFS was dose-dependent, with overall 8.8 months (95% CI, 5.6 months to 11.6 months), 12.1 months (95% CI, 8.8 months to 12.3 months) at a dose of 450 × 10^6^ cells, and 20.2 months (95% CI, 12.3 months to not reached) in patients with CR or stringent CR [96]. Interestingly, after disease progression, 28 patients were retreated with the CAR T-cell product. Of those, 21% showed a second response [96]. Severe AEs grade 3 or 4 occurred in 99% of all patients [96]. Most grade 3 or grade 4 toxicities were hematologic events, including neutropenia (89%), anemia (60%), and thrombocytopenia (52%) [96]. In contrast to anti-CD19 CAR T-cell products, severe CRS or ICANS were quite uncommon and were observed in no more than 6% of all patients [96].

These promising results led to a multicenter, randomized, open-label, phase 3 study, investigating the safety and efficacy of idecabtagene vicleucel compared to standard regimens in patients with r/r MM (NCT03651128—KarMMa-3) [93]. Overall, 386 patients were assigned in a 2:1 ratio to receive idecabtagene vicleucel or a standard therapy regimen [97].

At a median follow-up of 18.6 months, the PFS was significantly higher in the idecabtagene vicleucel group at 13.3 months as compared to 4.4 months in the standard treatment group [97]. In addition, the OR was significantly higher in the CAR T-cell group with 71% (95% CI, 66–77%) as compared to 42% (95% CI, 33–50%) with HR 3.47 (95% CI, 2.24–5.39; *p* < 0.001) in the standard treatment group [97].

Thus, the FDA and EMA approved idecabtagene vicleucel for heavily pretreated adult patients with r/r MM, making it the first approved anti-BCMA-CAR T-cell product [15,21].

In addition, on January 26, 2024 the Committee for Medicinal Products for Human Use (CHMP) of the EMA has recommended marketing authorization approval of idecabtagene vicleucel for the treatment of adult patients with r/r MM who have received at least two prior therapies, including an immunomodulatory agent (IMiD), a proteasome inhibitor (PI), and an anti-CD38 monoclonal antibody. Recommendation for approval was based on the phase 3 KarMMa-3 trial in which idecabtagene vicleucel demonstrated superiority over standard regimens, significantly improved PFS and a well-established safety profile with mostly low-grade occurrences of CRS and neurotoxicity (NCT03651128—KarMMa-3) [93].

### 4.5. Lisocabtagene Maraleucel (Breyanzi^®^)

Lisocabtagene maraleucel (formerly known as JCAR017) is an autologous anti-CD19 CAR T-cell product, which was developed for the treatment of CD19-positive B-cell malignancies [106]. The second-generation anti-CD19 CAR T receptor construct consists of an scFv of the CD19-specific mAb FMC63, a 4-1BB costimulatory domain, and an intracellular CD3ζ signaling domain [142]. In contrast to other anti-CD19 CAR T-cell products, the manufacturing process of lisocabtagene maraleucel involves a selection of CD8+ and CD4+ T-cells from the leukapheresis material, followed by independent CD8+ and CD4+ T-cell activation, transduction, expansion, formulation, and cryopreservation [143]. Lisocabtagene maraleucel is administered at equal target doses of CD8+ and CD4+ CAR T-cells and is given as two separate infusions, sequentially [106,143].

The safety, pharmacokinetics, and anti-tumor activity of lisocabtagene maraleucel in adult patients with r/r aggressive B-cell lymphoma were investigated in a phase 1 study (NCT02631044—TRANSCEND-NHL-001) [98,106,107]. In this study, eligible adult patients had PET-positive r/r DLBCL, high-grade B-cell lymphoma with rearrangements in MYC and either BCL2, BCL6, or both (double-hit or triple-hit lymphoma), PMBCL, or FL grade 3B [98,106]. All patients had received ≥2 previous lines of systemic treatment with subsequent relapse (previous autologous or allogeneic HSCT possible) [98,106,107]. Overall, 344 patients underwent leukapheresis for CAR T-cell production. CAR T-cells were administered to 294 (85%) patients: 269 received lisocabtagene maraleucel and 25 received a non-conforming CAR T-cell product [106]. Overall, 256 patients were included in the efficacy analysis. The OR rate was 73%, of whom 53% achieved a CR [106]. These good response rates were accompanied also by long-lasting effects, with a median DOR that was not reached (95% CI, 8.6 months—not reached) at a median follow-up of 12.0 months. The estimated DOR rate for 1 year was 55% (95% CI, 46.7–62.0%) for all participants and 65% (95% CI, 56.2–72.8%) in the CR group [106]. Of note, grade 3 or 4 CRS occurred in only 2% of all patients and ICANS in only 10% of all patients [106]. The most common grade ≥3 AEs were related to hematotoxicity, with neutropenia occurring in 60%, anemia in 37%, and thrombocytopenia in 27% [106,107].

In a phase 2 multicenter study to determine the efficacy and safety of lisocabtagene maraleucel, 74 patients underwent leukapheresis and 61 received the CAR T-cell product (NCT03483103—TRANSCEND-PILOT-017006) [102,108]. All patients had r/r large B-cell lymphoma and PET-positive disease and were not intended for high-dose chemotherapy and HSCT [108]. The median age was 74 years, and 26% had an Eastern Cooperative Oncology Group (ECOG) performance status of 2 [108]. The ORR was 80% (95% CI, 68–89%), with a CRR of 54% (95% CI, 41–67%) and PRR of 26% (95% CI, 16–39%) [108]. At a median follow-up of 13.0 months, the median PFS was 9.03 months, and at a median follow-up of 17.6 months, the median OS was not reached (95% CI, 17.28 months—not reached) [108]. The median DOR for patients with CR was 21.65 months (95% CI, 12.09 months—not reached) [108]. Notably, grade ≥ 3 CRS and ICANS occurred in only 2% and 5%, respectively [108]. These results suggested that CAR T-cell therapy is feasible in elderly and fragile patients, who are not eligible for high-dose chemotherapy and HSCT, and that CAR T-cell therapy leads to strong responses with long-lasting remissions [108].

In a global, randomized, multicenter phase 3 trial, lisocabtagene maraleucel was compared to SOC in adult patients with high-risk, second-line, transplant-eligible r/r aggressive B-cell NHL (NCT03575351—TRANSFORM) [105,109]. With a median follow-up of 17.5 months, the primary endpoint EFS was not reached for the CAR T-cell product and was 2.4 months for SOC [109]. The CRR was 74% for lisocabtagene maraleucel as compared to 43% for SOC (*p* < 0.0001); the median PFS was not reached for lisocabtagene maraleucel as compared to 6.2 months for SOC. In addition, the median OS was not reached (95% CI, 29.5 months to not reached) for lisocabtagene maraleucel as compared to 29.9 months (95% CI, 17.9 months to not reached) for SOC (*p* = 0.0987). There was no significant difference in the OS, which was due to a limited number of events (deaths). In fact, 66% of the patients in the SOC arm crossed over and received lisocabtagene maraleucel [109]. As with previous trials, the incidence of CAR T-cell-related AEs was well manageable, and the frequency of grade ≥3 CRS or ICANS was quite low (6% and 21%, respectively) [109].

These results led to the approval of lisocabtagene maraleucel by the FDA and EMA (Table 2).

### 4.6. Ciltacabtagene Autoleucel (Carvykti^®^)

Ciltacabtagene autoleucel is a CAR T-cell product developed for the treatment of r/r MM [14,20,113,144]. The CAR T-cell product consists of two BCMA-targeting nanobody heavy chains (VHH), a 4-1BB costimulatory domain, and a CD3ζ signaling domain [79,113,145]. Thus, ciltacabtagene autoleucel is able to bind two different BCMA epitopes [113].

In a single-arm, open-label, phase 1b/2 study, the safety and efficacy of ciltacabtagene autoleucel was investigated in patients with r/r MM (NCT03548207—CARTITUDE-1) [110,114]. Overall, 113 patients were enrolled and underwent leukapheresis [110,114]. Due to disease progression, death, or study withdrawal, 14% of the patients did not receive the final CAR T-cell product [113]. The ORR at a median follow-up of 12.4 months was 97% (95% CI, 91.2–99.4%). Overall, 67% of the patients developed a stringent CR [113]. The median PFS was not reached (95% CI, 16.8 months—not reached), and the overall 12-month PFS rate was 77% (95% CI; 66.0–84.3%) [113]. The 12-month OSR was 89% [113]. Hematological toxicities were the most common AEs, particularly cytopenias, whereas grade ≥3 CRS (4%) and ICANS (2%) were uncommon. Unfortunately, one patient subsequently died as a result of CRS and hemophagocytic lymphohistiocytosis [113,114]. Of note, besides ICANS, other neurotoxicities occurred in 12% of the patients, and one patient died from grade 5 neurotoxicity, whereas four patients died due to other reasons, so a further evaluation of the neurotoxicity outcome was not possible [113]. The CARTITUDE-1 trial proved that ciltacabtagene autoleucel leads to an early, deep, and durable response, with an ORR of 98% at two years, and did not reach median DOR and median PFS [114,115].

A phase 2 study of ciltacabtagene autoleucel investigated the overall MRD negativity rate (NCT04133636—CARTITUDE-2) [111,115]. In this study, patients with r/r MM were divided into several subgroups, and the response to treatment with ciltacabtagene autoleucel was evaluated [115]. In patients who had previously received a BCMA-targeting drug (e.g., belantamab mafodotin), ciltacabtagene autoleucel induced a MRD negativity rate of 70%, with an ORR of 60%, a median DOR of 11.5 months, and a median PFS of 9.1 months at a median follow-up of 11.3 months [115]. These results suggest that patients who have been treated previously with an anti-BCMA-therapy can still benefit from therapy with ciltacabtagene autoleucel [115].

To investigate ciltacabtagene autoleucel in earlier treatment lines in patients with lenalidomide-refractory disease, a randomized, phase 3, open-label trial was conducted (NCT04181827—CARTITUDE-4) [112,146]. All patients had received one to three previous treatment lines. Overall, 419 patients were enrolled and 208 were treated with ciltacabtagene autoleucel as compared to 211 who received SOC treatment [146]. At a median follow-up of 15.9 months, the median PFS was not reached in the ciltacabtagene autoleucel group as compared to 11.8 months in the SOC group. The PFS at 12 months was 75.9% (95% CI, 69.4% to 81.1%) after treatment with ciltacabtagene autoleucel as compared to 48.6% (95% CI, 41.5% to 55.3%) after treatment with SOC. Ciltacabtagene autoleucel induced a higher OS rate (84.6% vs. 67.3%), a higher CRR (73.1% vs. 21.8%), and a higher rate of MRD negativity (60.6% vs. 15.6%) [146]. Treatment with ciltacabtagene autoleucel was associated with typical AEs, e.g., hematologic toxicities, CRS, and ICANS, but fortunately, grade ≥ 3 CRS (1.1%, no grade 5) and ICANS (0%) were uncommon [146].

Thus, the FDA approved ciltacabtagene autoleucel in March 2021, and the EMA granted marketing authorization in May 2022. 

In addition, on 22 February 2024, the CHMP of the EMA adopted an extension to the existing indication to include treatment of adult patients with r/r MM who have received at least one prior therapy, including an immunomodulatory agent and a proteasome inhibitor, have demonstrated disease progression on the last therapy, and are refractory to lenalidomide.

## 5. Treatment-Related Adverse Events

CAR T-cell therapy has improved the response to treatment and outcome in many hematologic malignancies [147]. But these strong effects come with a cost: Treatment-related AEs are frequent and need to be addressed by a well-trained team of experts [147]. Over the last years, in clinical trials, but also in clinical routine, there has been a better understanding of these, in part specific side effects, and guidelines for prevention and prophylaxis have been published [148,149,150,151]. This has led to a remarkable improvement in management and prognosis, and fatal outcomes have become rare events [148,149,150,151]. Nevertheless, every patient needs to be evaluated properly ahead of CAR T-cell therapy, and risk factors, e.g., high tumor burden, uncontrolled disease, secondary diagnoses, and performance status (e.g., ECOG score), have to be taken into account [151]. A thorough examination and interpretation of these and other baseline parameters (such as lactate dehydrogenase, c-reactive protein (CRP), lung function, heart function) can reduce the risk of serious and fatal side effects, sufficiently [150,151].

The following overview highlights the most common CAR T-cell-specific AEs and gives some information about prophylaxis and treatment.

### 5.1. Immune Effector Cell-Associated Hematotoxicity (ICAHT)/Hemophagocytic Lymphohistiocytosis (HLH)/Macrophage Activation Syndrome (MAS) after CAR T-Cell Therapy

CAR T-cell therapy is associated with hematologic toxicities, which can be severe and long-lasting [149]. Hematologic toxicity represents the most common grade ≥3 AEs after CAR T-cell therapy and can pave the way to serious infections, which are then major drivers of morbidity and non-relapse mortality after therapy. Therefore, the term “immune effector cell-associated hematotoxicity” (ICAHT) has been introduced for further investigation and the development of treatment recommendations [149,152,153,154]. Severe cytopenias occur after CAR T-cell therapy regardless of the specific target antigen and are described across various malignancies [149]. The underlying pathophysiologic mechanisms are still under investigation, but growing evidence shows that patients’ individual hematopoiesis ahead of CAR T-cell infusion and inflammatory stress are most relevant [149,155,156]. In 2023, the European Hematology Association (EHA) and the European Society for Blood and Marrow Transplantation (EBMT) published consensus recommendations for the treatment of ICAHT, based on an international expert committee [154]. Based on an international survey, the expert panel divided ICAHT into an early form (occurrence within 30 days after CAR T-cell infusion) and a late form (occurrence beyond day +30 after CAR T-cell infusion) [154]. A grading system based on the neutrophil count was proposed, which includes the onset and duration as well as the severity of neutropenia [154]. Risk factors associated with the occurrence of ICAHT are disease-related features (e.g., disease burden), previous therapies (e.g., number of treatment lines, used drugs), bone marrow function (e.g., bone marrow infiltration, clonal hematopoiesis of indeterminate potential), inflammatory markers (CRP, ferritin), and CAR T-cell product specificities (e.g., costimulatory domain, type of construct) [153,154]. The “CAR-HEMATOTOX” score can help to identify patients at a high risk for prolonged neutropenia and can be calculated ahead of lymphodepleting conditioning, although it features only a limited positive predictive value [149,153,154].

Besides ICAHT, which is relatively common, the incidence of hemophagocytic lymphohistiocytosis (HLH) ranges from 1% to 3.4% [157,158]. HLH is a serious inflammatory syndrome characterized by elevated blood ferritin levels, coagulatory dysfunction, hepatic impairment, and cytopenia [159]. The American Society of Transplantation and Cellular Therapy (ASTCT) composed a working group of 30 experts to provide a clinical guideline for the recognition and treatment of the newly termed “Immune Effector Cell-associated HLH-like syndrome” (IEC-HS) [159]. The ASTCT developed an IEC-HS grading system and provides clinicians with helpful treatment recommendations, although admitting the lack of clinical evidence and the need for prospective clinical examinations [159].

In summary, due to the lack of prospective clinical trials, treatment recommendations depend mainly on expert opinions [149,159]. There are no specific recommendations for the transfusion of blood products available. Infectious prophylaxis is similar to recommendations for allogeneic HSCT [149]. The expert panel of EHA/EBMT proposes the prophylactic use of G-CSF for patients with a high-risk profile for ICAHT, whereas thrombopoietin (TPO) agonists are considered primarily in the context of prolonged and late-onset thrombocytopenia [149]. However, data on supporting the use of TPO agonists are very limited [149]. In situations of prolonged cytopenia, usually characterized by unresponsive neutropenia to G-CSF stimulation beyond day +14 after CAR T-cell infusion, or sustained anemia and thrombocytopenia, the transfusion of autologous hematopoietic stem cells (HSCs) should be considered [160,161]. Allogeneic HSCT remains an option when autologous HSCs are unavailable or in cases of treatment failure. Nevertheless, allogeneic HSCT is not considered as a routine procedure and needs to be discussed for each patient, individually [154].

### 5.2. Cytokine Release Syndrome (CRS)

Besides hematotoxicity, CRS is the most common AE after CAR T-cell infusion [151,158,162]. It is triggered by the activation of T-cells, which release cytokines and other mediators to activate surrounding bystander immune cells [158,162]. Patients typically experience constitutional symptoms, e.g., fever, headache, and myalgias, but serious and life-threatening complications, such as hypoxia, hypotension, and shock, may also occur [158]. Usually, CRS symptoms develop during the first week after CAR T-cell infusion, with a peak of severity about 1–2 weeks after administration [151,158,162]. Therefore, patients have to be monitored systematically, and precautions have to be taken ahead of therapy [148,151]. There are several grading systems available [158,163,164,165], making comparisons between clinical studies and the development of treatment guidelines difficult [158]. The ASTCT initiated a harmonization meeting, including members of the Center for International Blood and Marrow Transplant Research (CIBMTR), the American Society of Hematology (ASH), and the National Cancer Institute (NCI) [160]. This consensus on CRS grading divides CRS into five different grades, depending on clinical symptoms, like fever, hypotension, and hypoxia (Table 4) [148].

There are several CRS definitions and grading systems in use, making comparisons between clinical studies and the development of treatment guidelines difficult. The ASTCT CRS Consensus Grading system comprises three clinical parameters (fever, hypotension, hypoxia) that are robust and can be easily evaluated.

The EBMT and the Joint Accreditation Committee of International Society for Cell & Gene Therapy and EBMT (JACIE) and the EHA have established best practice recommendations for the management of CAR T-cell therapy [150]. Based on the above-mentioned ASTCT consensus criteria, an algorithm was created for each CRS grade, giving detailed information on medical action and intervention [150]. Depending on the severity and duration of CRS, the recommendation comprises the use of tocilizumab, glucocorticoids, and supportive treatment and, in serious situations, transfer to an intensive care unit [150,151].

### 5.3. Immune Effector Cell-Associated Neurotoxicity Syndrome (ICANS)

ICANS is a common adverse event after CAR T-cell therapy and may manifest, e.g., as encephalopathy, aphasia, lethargy, disorientation, agitation, or cerebral edema [148,151,158,166]. These neurologic symptoms may occur simultaneously or after CRS [148,167]. The physiologic processes behind ICANS remain generally unclear, and there are currently no established diagnostic tools available which would help to predict the onset and severity of ICANS ahead of CAR T-cell therapy [148,167]. Similarly to CRS, the ASTCT developed a consensus ICANS grading system (Table 5) [148].

The working group introduced the immune effector cell-associated encephalopathy (ICE) score (Table 6) to objectively evaluate patients’ neurologic constitution [148]. Depending on the ICE score results, the ICANS score is determined and divided into five different grades [148]. This consensus definition provides a helpful tool to evaluate ICANS and it helps to define, analyze, and compare ICANS in prospective clinical trials.

Importantly, the ICE score assessment is a valuable analytic tool for the supervision of adult patients during CAR T-cell therapy. For <12 yo children, the ASCTC recommends the use of the “Cornell Assessment of Pediatric Delirium” (CAPD) questionnaire [148]. Therefore, a pediatrician evaluates behavioral aspects of the child, activity level, and response to interactions [148].

Treatment recommendations have been developed by the EBMT, JACIE, and EHA [150]. The specific treatment algorithm, which is adapted for the ICANS grading system by ASTCT [148], comprises the use of dexamethasone and methylprednisolone [150]. The therapeutic role for tocilizumab in the context of ICANS is unclear, and its use is therefore not recommended [150,151,167].

## 6. CAR T-Cell Therapy in Solid Oncology

In contrast to the phenomenal success of CAR T-cell therapy in hematologic malignancies, CAR T-cells have largely failed in solid oncology, so far [78,80]. Major challenges include on-target off-tumor toxicity, CRS, tumor antigen heterogeneity, and the immunosuppressive tumor microenvironment (TME) [76,80,168].

Especially the TME causes some obstacles which are difficult to overcome [76,80,169]. A tumor represents a hostile microenvironment for T lymphocytes [169]. The reduced expression of adhesion molecules on endothelial cells hampers adhesion and trafficking of lymphocytes into the tumor [169]. Besides physical barriers, hypoxia and alterations in the energy metabolism of tumor cells lead to an uncomfortable area that limits the survival and function of different immune cells because of a lack of nutrition and oxygen [169]. Additionally, tumor cells and the surrounding bystander cells (e.g., regulatory T-cells) actively inhibit immune cell function by secreting immunosuppressive cytokines, leading to anergy and the apoptosis of tumor-infiltrating lymphocytes and immune effector cells [76,80,166].

Another hurdle is the heterogenous expression of so-called tumor-specific antigens in solid tumors and, as a result, on-target off-tumor activity [76,77]. Anti-CD19 CAR T-cells and anti-BCMA CAR T-cells are effective in the treatment of B-cell malignancies and MM because of the highly specific expression of these antigens on B-cells and plasma cells, respectively. But this effect is not tumor-specific, as these CAR T-cell products eliminate every cell expressing these antigens on its surface, without discriminating between normal and abnormal, cancerous cells [77]. The off-tumor activity, which leads to B-cell or plasma cell aplasia, can be tolerated in these instances, and the positive treatment effect outweighs this adverse reaction [77]. Although tumors often show a higher expression of certain antigens, these antigens are also present on healthy cells in different tissues. Therefore, CAR T-cells can be directed against healthy tissue, which can lead in these situations to intolerable toxicities, which limits the use of these CAR T-cell products [76,77]. Besides these challenges, solid tumors are typically very heterogenous, meaning that the expression of certain antigens varies within the tumor, leading to a negative selection of tumor cells not expression the target antigen [77,79]. This antigen escape also plays a major role of resistance in hematologic malignancies, where antigen loss under CAR T-cell therapy is a well-known phenomenon [76,79]. Treatment-related effects, which are eminent for CAR T-cell function, can also be dangerous if they appear in certain locations. In clinical trials for tumors of the central nervous system (CNS), distinct from CRS and ICANS, tumor inflammation-associated neurotoxicity (TIAN) is a serious AE which limits the therapeutic use of CAR T-cells [170]. This is a dilemma for the treatment of CNS malignancies: on one hand, CAR T-cells shall deliver their anti-tumor activity at the site of the tumor (on-target effect); on the other hand, the pharmacologic mechanism, which is the immune-mediated inflammation against the tumor, leads to side effects, which can be fatal in the CNS [170].

To date, there are no CAR T-cell products approved for the treatment of solid tumors, and there is still much research needed to overcome the above-mentioned obstacles and limitations.

## 7. Obstacles and Limitations

CAR T-cells combine two aspects of the adoptive immune system: the antigen specificity of an antibody binding domain and the cytotoxic and immune modulating activity of the T-cell system [77,80]. This makes CAR T-cell products a powerful tool for the eradication of specific target cells [77]. Hence, these two features are also responsible for central obstacles and limitations [77,80]. A key limitation for CAR T-cell design is identifying a targetable and tumor-specific antigen [77,80]. Most antigens of interest are not tumor-specific in a proper sense, meaning that these antigens are also expressed by healthy cells. Additionally, due to tumor heterogeneity, tumor cells often show different expression levels of an antigen of interest, and in the context of tumor evolution, antigen loss is often experienced [76,77,80]. Besides this fundamental problem, there are many more challenges, e.g., CAR T-cell expansion, persistence, tumor infiltration, TME, and serious AEs, which potentially limit the use of CAR T-cells [76,77,80].

## 8. Conclusions

Over the last years, CAR T-cell therapy has become a new treatment option for certain hematologic malignancies, and many patients have benefited from its use. Besides its success in B-cell and plasma cell malignancies, there have been reports about the efficacy of CAR T-cells in different areas of medicine, e.g., solid oncology, autoimmune diseases, and infectious diseases [76,77,80]. Nevertheless, there are limitations and obstacles that must be taken seriously, and further research is needed. Severe, sometimes life-threatening side effects, on-target off-tumor effects, tumor heterogeneity, antigen escape, and many more challenges remain to be solved [76,77,80]. Finally, emerging data suggest that there is a certain risk of T-cell malignancies after CAR T-cell therapy [171]. However, existing data from follow-up studies suggest a low risk compared with other cancer treatments. Thus, the benefits of CAR T-cells should not be withheld when it appears to be the best option available. Nevertheless, patients and clinical trial participants receiving treatment with these products should be monitored for life for new malignancies. Besides these medical and scientific difficulties, therapy-related costs are also worth mentioning and are a relevant burden for the health care system. However, the manufacturing of CAR T-cell products will probably become less expensive over the next years. Due to new competitors and the expiry of protecting patents, manufacturing capacities will increase, which will have a relevant impact on supply and demand. This will probably lead to a significant price reduction.

Emerging new technologies, improvements in CAR design, and the combination of different treatment modalities will be helpful in overcoming these difficulties. CAR T-cells’ triumph has just begun.

## Figures and Tables

**Table 1:** Milestones of CAR T-cell development.

1980s	First reports on chimeric antigen receptor T-cells (e.g., Kuwana et al. [33], Becker et al. [34], Eshhar et al. [35,36], Goverman et al. [37])
1990s	“First-generation” CAR T-cells with scFv (Eshhar et al. [38])
2000s	First clinical trials of “first-generation” CAR T-cells in metastatic solid tumors (e.g., Kershaw et al. [39], Park et al. [40], Lamers et al. [41]); development of “second-generation” CAR T-cells (e.g., Imai et al. [42], Maher et al. [43])
2010s	Successful use of “second-generation” CAR T-cells in several clinical trials [44,45,46,47,48,49,50,51,52,53,54,55,56,57]
2017	FDA approval of first CAR T-cell product (tisagenlecleucel)

**Table 2:** Overview of the approved CAR T-cell products with indications.

Trade Name	Generic Name	Targeted Antigen	Costimulatory Domains	FDA Approval	EMA Approval
KYMRIAH^®^	Tisagen- lecleucel	CD19	4-1BB	- Patients ≤ 25 yo with B-cell precursor ALL that is refractory or in second or later relapse. - Adult patients with r/r LBCL after two or more lines of systemic therapy, including DLBCL not otherwise specified, HGBL, and DLBCL arising from FL. Limitations of Use: KYMRIAH is not indicated for treatment of patients with primary central nervous system lymphoma. - Adult patients with r/r FL after two or more lines of systemic therapy [17].	- Patients ≤ 25 yo with B-cell ALL that is refractory, in relapse post-transplant or in second or later relapse. - Adult patients with r/r DLBCL after two or more lines of systemic therapy. - Adult patients with r/r FL after two or more lines of systemic therapy [23].
YESCARTA^®^	Axicabtagene ciloleucel	CD19	CD28	- Adult patients with LBCL that is refractory to first-line chemoimmunotherapy or that relapses within 12 months of first-line chemoimmunotherapy. - Adult patients with r/r LBCL after two or more lines of systemic therapy, including DLBCL not otherwise specified, PMBCL, HGBL, and DLBCL arising from FL. - Adult patients with r/r FL after two or more lines of systemic therapy [12].	- Adult patients with DLBCL and HGBL that relapses within 12 months from completion of, or is refractory to, first-line chemoimmunotherapy. - Adult patients with r/r DLBCL and PMBCL, after two or more lines of systemic therapy. - Adult patients with r/r FL after three or more lines of systemic therapy [18].
TECARTUS^®^	Brexucabtagene autoleucel	CD19	CD28	- Adult patients with r/r MCL. - Adult patients with r/r B-cell precursor ALL [13].	- Adult patients with r/r MCL after two or more lines of systemic therapy including a Bruton’s tyrosine kinase (BTK) inhibitor. - Patients ≥ 26 yo with r/r B-cell precursor ALL [19].
ABECMA^®^	Idecabtagene vicleucel	BCMA	4-1BB	- Adult patients with r/r MM after four or more prior lines of therapy, including an immunomodulatory agent, a proteasome inhibitor, and an anti-CD38 monoclonal antibody [15].	- Adult patients with r/r MM who have received at least three prior therapies, including an immunomodulatory agent, a proteasome inhibitor, and an anti-CD38 antibody and have demonstrated disease progression with the last therapy [21].
BREYANZI^®^	Lisocabtagene maraleucel	CD19	4-1BB	- Adult patients with LBCL, including DLBCL not otherwise specified (including DLBCL arising from indolent lymphoma), HGBL, PMBCL, and FL grade 3B, who have the following: - A refractory disease to first-line chemoimmunotherapy or relapse within 12 months of first-line chemoimmunotherapy; - A refractory disease to first-line chemoimmunotherapy or a relapse after first-line chemoimmunotherapy and are not eligible for hematopoietic stem cell transplantation (HSCT) due to comorbidities or age; - An r/r disease after two or more lines of systemic therapy [16].	- Adult patients with DLBCL, HGBL, PMBCL, and FL grade 3B, who relapsed within 12 months from completion of, or are refractory to, first-line chemoimmunotherapy. - Adult patients with r/r DLBCL, PMBCL, and FL grade 3B, after two or more lines of systemic therapy [22].
CARVYKTI^®^	Ciltacabtagene autoleucel	BCMA	4-1BB	- Adult patients with r/r MM after four or more prior lines of therapy, including a proteasome inhibitor, an immunomodulatory agent, and an anti-CD38 monoclonal antibody [14].	- Adult patients with r/r MM, who have received at least three prior therapies, including an immunomodulatory agent, a proteasome inhibitor, and an anti-CD38 antibody and have demonstrated disease progression with the last therapy [20].

Abbreviations: ALL, acute lymphoblastic leukemia; DLBCL, diffuse large B-cell lymphoma; FL, follicular lymphoma; HGBL, high-grade B-cell lymphoma; LBCL, large B-cell lymphoma; MCL, mantle cell lymphoma; MM, multiple myeloma; PMBCL, primary mediastinal B-cell lymphoma; r/r, relapsed/refractory. Treatment indications of the U.S. Food and Drug Administration (FDA) and the European Medicines Agency (EMA) are generally comparable but differ in some minor aspects. Therefore, treating physicians have to be aware of the marketing authorization of their specific region (February 2024).

**Table 3:** Overview of the most relevant clinical trials for treatment approval.

Trade Name Generic Name	Most Relevant Clinical Trials	Summary of Most Relevant Results
KYMRIAH^®^ Tisagenlecleucel	NCT02435849—Study of Efficacy and Safety of CTL019 in Pediatric ALL Patients (ELIANA) [44] NCT02445248—Study of Efficacy and Safety of CTL019 in Adult DLBCL Patients (JULIET) [45] NCT02228096—Study of Efficacy and Safety of CTL019 in Pediatric ALL Patients (ENSIGN) [46] NCT03568461—Efficacy and Safety of Tisagenlecleucel in Adult Patients With r/r Follicular Lymphoma (ELARA) [47] NCT01029366—CART19 to Treat B-Cell Leukemia or Lymphoma That Are Resistant or Refractory to Chemotherapy [48] NCT01747486—Dose Optimization Trial of CD19 Redirected Autologous T Cells [49] NCT02030847—Study of Redirected Autologous T Cells Engineered to Contain Anti-CD19 Attached to TCR and 4-1BB Signaling Domains in Patients With Chemotherapy Resistant or Refractory Acute Lymphoblastic Leukemia [50] NCT02030834—Phase IIa Study of Redirected Autologous T Cells Engineered to Contain Anti-CD19 Attached to TCRz and 4-Signaling Domains in Patients With Chemotherapy Relapsed or Refractory CD19+ Lymphomas [51]	ELIANA: primary end point: ORR 82.3% (95% CI, 72.1 to 90.0); secondary end points: BOR 82.1% (95% CI, 70.8 to 90.4); BOR with MRD negative BM 81.0% (95% CI, 70.6 to 89.0); total SAEs 79.75%, CRS 63.29%, ICANS: 3.75% [44] JULIET: primary end point: ORR 52% (95% CI, 41 to 62); secondary end points: 12-month median RFS 65%; CRS ≥ 3: 22%, ICANS ≥ 3: 12% [52] ENSIGN: primary end point ORR: 70.3% (95% CI, 57.6 to 81.1); secondary end points: median EFS 15.6 months (95% CI, 6.4 to NA), median OS 29.9 months (95% CI, 15.1 to 42.4); total SAEs 81.25%, CRS 64.06%, ICANS 6.25% [46] ELARA: primary end point CRR: 69.1% (95% CI, 58.8 to 78.3); secondary end points: ORR 86.2 (95% CI, 77.5 to 92.4), total SAEs 43.3%, CRS 19.59%, ICANS 1.03% [47]
YESCARTA^®^ Axicabtagene ciloleucel	NCT02348216—Study Evaluating the Safety and Efficacy of KTE-C19 in Adult Participants With Refractory Aggressive Non-Hodgkin Lymphoma (ZUMA-1) [53] NCT03105336—A Phase 2 Multicenter Study of Axicabtagene Ciloleucel in Subjects With r/r Indolent Non-Hodgkin Lymphoma (ZUMA-5) [54] NCT03153462—Axicabtagene Ciloleucel Expanded Access Study (ZUMA-9) [55] NCT03761056—Study to Evaluate the Efficacy and Safety of Axicabtagene Ciloleucel as First-Line Therapy in Participants With High-Risk Large B-Cell Lymphoma (ZUMA-12) [56] NCT03391466—Study of Effectiveness of Axicabtagene Ciloleucel Compared to Standard of Care Therapy in Patients With r/r Diffuse Large B Cell Lymphoma (ZUMA-7) [57]	ZUMA-1: primary end points: cohort 1 ORR 82% (95% CI, 71 to 90), cohort 2 ORR 83% (95% CI, 63 to 95), cohort 3: CRS ≥ 3: 3%, ICANS ≥ 3: 39%, ICANS 5: 3%, cohort 4: CRS ≥ 3: 2%, ICANS ≥ 3: 17%, cohort 5: CRS ≥ 3: 2%, ICANS ≥ 3: 12%, cohort 6: CRS ≥ 3: 0%, ICANS ≥ 3: 15%; total SAEs 71.43% [53] ZUMA-5: primary end point: ORR 94% (95% CI, 88–97) in FL and 77% (95% CI, 59–90) in MZL; key secondary end points: median PFS 40.2 months in FL, not reached in MZL; FL: total SAEs 11%, CRS ≥ 3: 0%, ICANS ≥ 3: 0%; MZL: total SAEs 14%, CRS ≥ 3: 11%, ICANS ≥ 3: 4% [81] ZUMA-12: primary end point: CRR 78% (95% CI, 62 to 90); secondary end points: ORR 89% (95% CI, 75 to 97), median OS 24.5 months (95% CI, 3.6 to 24.5), total SAEs 45% [56] ZUMA-7: primary end point: 25-month follow-up: EFS 8.3 vs. 2.0 months, HR 0.4, (95% CI, 0.31 to 0.51, *p* < 0.001); secondary end points: ORR 83% vs. 50% (*p* < 0.001), CR 65% vs. 32%, total SAEs 91% vs. 83%; CRS ≥ 3: 6% vs. 0%, neurologic events ≥ 3: 21% vs. 1% [82]
TECARTUS^®^ Brexucabtagene autoleucel	NCT02601313—Study of Brexucabtagene Autoleucel (KTE-X19) in Participants With r/r Mantle Cell Lymphoma (Cohort 1 and Cohort 2) (ZUMA-2) [83] NCT02614066—A Study Evaluating the Safety and Efficacy of Brexucabtagene Autoleucel (KTE-X19) in Adult Subjects With r/r B-precursor Acute Lymphoblastic Leukemia (ZUMA-3) [84] NCT02625480—Study Evaluating Brexucabtagene Autoleucel (KTE-X19) in Pediatric and Adolescent Participants With r/r B-precursor Acute Lymphoblastic Leukemia or r/r B-Cell Non-Hodgkin Lymphoma (ZUMA-4) [85] NCT03624036—Study to Evaluate the Safety and Tolerability of Brexucabtagene Autoleucel (KTE-X19) in People With r/r Chronic Lymphocytic Leukemia and Small Lymphocytic Lymphoma (ZUMA-8) [86] NCT04162756—Study of Brexucabtagene Autoleucel (KTE-X19) for the Treatment of Individuals With r/r MCL (ZUMA-18) [87]	ZUMA-2: primary end points: ORR 93% (95% CI, 84 to 98); secondary end points: CRR 67% (95% CI, 53 to 78), 12-month PFS 61% and OS 83%; CRS ≥ 3: 15%, ICANS ≥ 3: 31% [88] ZUMA-3: primary end points: CRR 70.9% (95% CI, 57 to 82); secondary end points: MRD negativity 76% (95% CI, 63 to 87), median DOR 12.8 months (95% CI, 8.7 to NA), median OS 18.2 (95% CI, 15.9 to NA), total SAEs: dose-dependent from 100% to 75%; CRS ≥ 3: 24%, ICANS ≥ 3: 25% [84,89,90] ZUMA-8: primary end point: dose limiting toxicities (DLTs): first-stage cohort 1: 0%, first-stage cohort 2: 0%, second-stage cohort 3: 33.3%, second-stage cohort 4A: 0%; secondary end points: ORR: first-stage cohort 1: 50% (95% CI, 11.8 to 88.2), first-stage cohort 2: 33% (95% CI, 0.8 to 90.6), second-stage cohort 3: 100% (95% CI, 29.2 to 100), second-stage cohort 4a: 0% (0.0 to 70.8) [86] ZUMA-18: ORR: 87%; median OS not yet reached at 33.5 months of follow-up; no new safety signals were detected
ABECMA^®^ Idecabtagene vicleucel	NCT03361748—Efficacy and Safety Study of bb2121 in Subjects With r/r Multiple Myeloma (KarMMa) [91] NCT03601078—An Efficacy and Safety Study of bb2121 in Subjects With r/r Multiple Myeloma and in Subjects With High-Risk Multiple Myeloma (KarMMa-2) [92] NCT03651128—Efficacy and Safety Study of bb2121 Versus Standard Regimens in Subjects With r/r Multiple Myeloma (RRMM) (KarMMa-3) [93] NCT02658929—Study of bb2121 in Multiple Myeloma [94] NCT02786511—Longterm Follow-up of Subjects Treated With bb2121 [95]	KarMMa: primary end point: ORR 73% (95% CO, 66 to 81; *p* < 0.001); secondary end points: CRR 33%, VGPR or better 52%, MRD negativity 26% (95% CI, 19 to 34), median DOR 10.7 months (95% CI, 9.0 to 11.3), median PFS 8.8 months (95% CI, 5.6 to 11.6), total SAEs 99%, CRS ≥ 3: 5%, ICANS ≥ 3: 3% [96] KarMMa-3: primary end point: at 18.6 months mPFS 13.3 vs. 4.4 months (HR 0,49, 95% CI, 0.38 to 0.65; *p* < 0.001); secondary end points: ORR 71% vs. 42% (*p* < 0.001), CR 39% vs. 5%; total SAE 93% vs. 75%, CRS ≥ 3: 5%, ICANS ≥ 3: 3% [97]
BREYANZI^®^ Lisocabtagene maraleucel	NCT02631044—Study Evaluating the Safety and Pharmacokinetics of JCAR017 in B-cell Non-Hodgkin Lymphoma (TRANSCEND-NHL-001) [98] NCT03484702—Trial to Determine the Efficacy and Safety of JCAR017 in Adult Participants With Aggressive B-Cell Non-Hodgkin Lymphoma (TRANSCENDWORLD) [99] NCT03744676—A Safety Trial of Lisocabtagene Maraleucel (JCAR017) for r/r B-cell Non-Hodgkin Lymphoma (NHL) in the Outpatient Setting (TRANSCEND-OUTREACH-007) [100] NCT03310619—A Safety and Efficacy Trial of JCAR017 Combinations in Subjects With r/r B-cell Malignancies (PLATFORM) [101] NCT03483103—Lisocabtagene Maraleucel (JCAR017) as Second-Line Therapy (TRANSCEND-PILOT-017006) [102] NCT03331198—Study Evaluating Safety and Efficacy of JCAR017 in Subjects With Relapsed or Refractory Chronic Lymphocytic Leukemia (CLL) or Small Lymphocytic Lymphoma (SLL) [103] NCT03743246—A Study to Evaluate the Safety and Efficacy of JCAR017 in Pediatric Subjects With r/r B-cell Acute Lymphoblastic Leukemia (B-ALL) and B-cell Non-Hodgkin Lymphoma (B-NHL) [104] NCT03575351—A Study to Compare the Efficacy and Safety of JCAR017 to Standard of Care in Adult Subjects With High-risk, Transplant-eligible Relapsed or Refractory Aggressive B-cell Non-Hodgkin Lymphomas (TRANSFORM) [105]	TRANSCEND-NHL-001: primary end point: ORR 73% (95% CI, 66.8 to 78.0); secondary end points: CRR 53% (95% CI, 46.8 to 59.4); 12-month DOR 54.7% (95% CI, 46.7 to 62.0), median PFS 6.8 months (95% CI, 3.3 to 14.1), 12-month OS 57.9% (95% CI, 51.3 to 63.8); total SAE 79%, CRS ≥ 3: 2%, ICANS ≥ 3: 10% [106,107] TRANSCEND-OUTREACH-007: primary end points: CRS ≥3: 0.0% (95% CI, 0.0 to 4.4), ICANS ≥3: 9.8% (95% CI, 4.3 to 18.3); secondary end points: total SAE: 74.4%, ORR 80.5% (95% CI, 70.3 to 88.4), CRR 53.7 (95% CI, 42.3 to 64.7), median DOR 14.75 months (95% CI, 5.03 to NA), median DOCR NA (95% CI, 16.59 to NA), median PFS 5.83 months (95% CI, 0.7 to 24.5), median OS 22.01 months (95% CI, 1.0 to 27.3) [100] TRANSCEND-PILOT-017006: primary end point: ORR 80.3% (95% CI, 68.2 to 89.4); secondary end points: total SAE 78.7%, CRR 54.1% (95% CI, 40.8 to 66.9), median DOR 23.26 months (95% CI, 6.24 to NA), median PFS 9.03 (95% CI, 4.17 to NA), median EFS 7.23 months (95% CI, 3.22 to 24.28), median OS NA (95% CI, 16.33 to NA), CRS ≥ 3: 2%, ICANS ≥ 3: 5% [102,108] TRANSFORM: primary end point: median EFS NA (95% CI, 9.5 to NA) vs. 2.4 months (95% CI, 2.2 to 4.9), HR 0.356 (0.243 to 0.522); secondary end points: CRR 68% (95% CI, 63.7 to 82.5) vs. 40% (95% CI, 33.2 to 54.2) *p* < 0.0001, median PFS NA (95% CI, 12.6 to NA) vs. 6.2 months (95% CI, 4.3 to 8.6), HR 0.400 (95% CI, 0.261–0.615) *p* < 0.0001, median OS NA (95% CI, 29.5 to NA) vs. 29.9 (95% CI, 17.9 to NA), HR 0.724 (95% CI, 0.443 to 1.183) *p* = 0.099, total SAE 85% vs. 81%, CRS ≥3: 1%, ICANS ≥ 3: 4% [105,109]
CARVYKTI^®^ Ciltacabtagene autoleucel	NCT03548207—A Study of JNJ-68284528, a Chimeric Antigen Receptor T Cell (CAR-T) Therapy Directed Against B-Cell Maturation Antigen (BCMA) in Participants With Relapsed or Refractory Multiple Myeloma (CARTITUDE-1) [110] NCT04133636—A Study of JNJ-68284528, a Chimeric Antigen Receptor T Cell (CAR-T) Therapy Directed Against B-cell Maturation Antigen (BCMA) in Participants With Multiple Myeloma (CARTITUDE-2) [111] NCT04181827—A Study Comparing JNJ-68284528, a CAR-T Therapy Directed Against B-cell Maturation Antigen (BCMA), Versus Pomalidomide, Bortezomib and Dexamethasone (PVd) or Daratumumab, Pomalidomide and Dexamethasone (DPd) in Participants With Relapsed and Lenalidomide-Refractory Multiple Myeloma (CARTITUDE-4) [112]	CARTITUDE-1: primary end point: ORR 97.9% (95% CI, 92.7 to 99.7); secondary end points: sCR 82.5% (95% CI, 73.4 to 89.4), MRD negative sCR 44.3% (95% CI, 34.2 to 54.8), 27-month OS 70.4%, total SAE 91%, CRS ≥ 3: 5.1% (one related death), ICANS ≥ 3: 12.3% (one related death) [113,114] CARTITUDE-2: primary end point: MRD negativity 35% (95% CI, 15.4 to 59.2); secondary end points: ORR 60.0% (95% CI, 36.1 to 80.9), median DOR 11.5 months (95% CI, 7.0 to NA), total SAE 95%, CRS ≥ 3: 0%, ICANS ≥ 3: 10% [115] CARTITUDE-4: primary end point: 12-month PFS 75.9% (95% CI, 69.4 to 81.1) vs 48.6% (95% CI, 41.5 to 55.3); secondary end points: ORR 84.6% vs. 67.3%, HR 2.2 (95% CI, 1.5 to 3.1) *p* < 0.001, CR or better 73.1% vs. 21.8%, HR 2.9 (95% CI, 2.3 to 3.7) *p* < 0.001; total SAE 96.6% vs. 94.2%, CRS ≥ 3: 1.1%, ICANS ≥ 3: 2.8% [115]

**Table 4:** ASTCT CRS Consensus Grading [148].

CRS	Grade 1	Grade 2	Grade 3	Grade 4	Grade 5
Fever (not attributable to other causes)	≥38 °C	≥38 °C	≥38 °C	≥38 °C	Death
With					
Hypotension	None	Not requiring vasopressors	Requiring a vasopressor with or without vasopressin	Requiring multiple vasopressors (excluding vasopressin)	Death
And/or					
Hypoxia	None	Requiring low-flow nasal cannula or blow-by	Requiring high-flow nasal cannula, facemask, nonrebreather mask, or venturi mask	Requiring positive pressure (e.g., CPAP, BiPAP, intubation, and mechanical ventilation)	Death

Abbreviations: BiPAP, bilevel positive airway pressure; CPAP, continuous positive airway pressure; CRS, cytokine release syndrome.

**Table 5:** ASTCT ICANS Consensus Grading for Adults [148].

Neurotoxicity	Grade 1	Grade 2	Grade 3	Grade 4
ICE score	7–9	3–6	0–2	0 Patient is unarousable and unable to perform ICE
Depressed level of consciousness	Awakens spontaneously	Awakens to voice	Awakens only to tactile stimulus	Patient is unarousable or requires vigorous or repetitive tactile stimuli to arouse (stupor or coma)
Seizure	NA	NA	Any clinical seizure, focal or generalized, which resolves rapidly or nonconvulsive seizures on EEG that resolve with intervention	Life-threatening prolonged seizure (>5 min) or repetitive clinical or electrical seizures without return to baseline in between
Motor findings	NA	NA	NA	Deep focal motor weakness such as hemiparesis or paraparesis
Elevated ICP/cerebral edema	NA	NA	Focal/local edema on neuroimaging	Diffuse cerebral edema on neuroimaging; decerebrate or decorticate posturing or cranial nerve VI palsy; papilledema; or Cushing’s triad

Abbreviations: EEG, electroencephalography; ICE, immune effector cell-associated encephalopathy; ICP, intracranial pressure. Immune effector cell-associated neurotoxicity is characterized by different mainly unspecific symptoms. The ASTCT ICANS Consensus Grading for Adults evaluates neurologic symptoms and the results of neuroimaging techniques.

**Table 6:** Immune effector cell-associated encephalopathy (ICE) score [148].

Ability	Points
Orientation	Ability to name the year, month, city, and hospital: 4 points
Naming	Ability to name 3 different objects: 3 points
Following commands	Ability to follow simple commands (e.g., “show me 2 fingers”): 1 point
Writing	Ability to write a certain sentence (e.g., “our national bird is the bald eagle”): 1 point
Attention	Ability to count backwards from 100 by 10 to 0: 1 point

Scoring: best result: 10 points, no impairment; worst result: 0 points, patient unarousable and unable to perform ICE assessment (grade 4 ICANS).

## References

[1] Riley R.S., June C.H., Langer R., Mitchell M.J. (2019). Delivery technologies for cancer immunotherapy. Nat. Rev. Drug Discov..

[2] Fenis A., Demaria O., Gauthier L., Vivier E., Narni-Mancinelli E. (2024). New immune cell engagers for cancer immunotherapy. Nat. Rev. Immunol..

[3] Waldman A.D., Fritz J.M., Lenardo M.J. (2020). A guide to cancer immunotherapy: From T cell basic science to clinical practice. Nat. Rev. Immunol..

[4] Fanouriakis A., Kostopoulou M., Alunno A., Aringer M., Bajema I., Boletis J.N., Cervera R., Doria A., Gordon C., Govoni M. (2019). 2019 update of the EULAR recommendations for the management of systemic lupus erythematosus. Ann. Rheum. Dis..

[5] Penack O., Marchetti M., Ruutu T., Aljurf M., Bacigalupo A., Bonifazi F., Ciceri F., Cornelissen J., Malladi R., Duarte R.F. (2020). Prophylaxis and management of graft versus host disease after stem-cell transplantation for haematological malignancies: Updated consensus recommendations of the European Society for Blood and Marrow Transplantation. Lancet Haematol..

[6] Chau C.H., Steeg P.S., Figg W.D. (2019). Antibody-drug conjugates for cancer. Lancet.

[7] Goebeler M.-E., Bargou R.C. (2020). T cell-engaging therapies—BiTEs and beyond. Nat. Rev. Clin. Oncol..

[8] Kolb H.J., Schattenberg A., Goldman J.M., Hertenstein B., Jacobsen N., Arcese W., Ljungman P., Ferrant A., Verdonck L., Niederwieser D. (1995). Graft-versus-leukemia effect of donor lymphocyte transfusions in marrow grafted patients. Blood.

[9] Kolb H.J., Mittermüller J., Clemm C., Holler E., Ledderose G., Brehm G., Heim M., Wilmanns W. (1990). Donor leukocyte transfusions for treatment of recurrent chronic myelogenous leukemia in marrow transplant patients. Blood.

[10] O’Neill A.T., Chakraverty R. (2021). Graft Versus Leukemia: Current Status and Future Perspectives. J. Clin. Oncol..

[11] Schmid C., Kuball J., Bug G. (2021). Defining the Role of Donor Lymphocyte Infusion in High-Risk Hematologic Malignancies. J. Clin. Oncol..

[12] U.S. Food and Drug Administration FDA Approval Axicabtagene Ciloleucel. https://www.fda.gov/vaccines-blood-biologics/cellular-gene-therapy-products/yescarta-axicabtagene-ciloleucel.

[13] U.S. Food and Drug Administration FDA Approval Brexucabtagene Autoleucel. https://www.fda.gov/vaccines-blood-biologics/cellular-gene-therapy-products/tecartus-brexucabtagene-autoleucel.

[14] U.S. Food and Drug Administration FDA Approval Ciltacabtagene Autoleucel. https://www.fda.gov/vaccines-blood-biologics/carvykti.

[15] U.S. Food and Drug Administration FDA Approval Idecabtagene Vicleucel. https://www.fda.gov/vaccines-blood-biologics/abecma-idecabtagene-vicleucel.

[16] U.S. Food and Drug Administration FDA Approval Lisocabtagene Maraleucel. https://www.fda.gov/vaccines-blood-biologics/cellular-gene-therapy-products/breyanzi-lisocabtagene-maraleucel.

[17] U.S. Food and Drug Administration FDA Approval Tisagenlecleucel. https://www.fda.gov/vaccines-blood-biologics/cellular-gene-therapy-products/kymriah-tisagenlecleucel.

[18] European Medicines Agency EMA Approval Axicabtagene Ciloleucel. https://www.ema.europa.eu/en/medicines/human/EPAR/yescarta.

[19] European Medicines Agency EMA Approval Brexucabtagene Autoleucel. https://www.ema.europa.eu/en/medicines/human/EPAR/tecartus.

[20] European Medicines Agency EMA Approval Ciltacabtagene Autoleucel. https://www.ema.europa.eu/en/medicines/human/EPAR/carvykti.

[21] European Medicines Agency EMA Approval Idecabtagene Vicleucel. https://www.ema.europa.eu/en/medicines/human/EPAR/abecma.

[22] European Medicines Agency EMA Approval Lisocabtagene Maraleucel. https://www.ema.europa.eu/en/medicines/human/EPAR/breyanzi.

[23] European Medicines Agency EMA Approval Tisagenlecleucel. https://www.ema.europa.eu/en/medicines/human/EPAR/kymriah.

[24] Mitchison N.A. (1955). Studies on the immunological response to foreign tumor transplants in the mouse. I. The role of lymph node cells in conferring immunity by adoptive transfer. J. Exp. Med..

[25] Barnes D.W., Loutit J.F. (1957). Treatment of murine leukaemia with X-rays and homologous bone marrow. II. Br. J. Haematol..

[26] Mathé G., Amiel J.L., Schwarzenberg L., Cattan A., Schneider M. (1965). Adoptive immunotherapy of acute leukemia: Experimental and clinical results. Cancer Res..

[27] Southam C.M., Brunschwig A., Levin A.G., Dizon Q.S. (1966). Effect of leukocytes on transplantability of human cancer. Cancer.

[28] Weiden P.L., Flournoy N., Thomas E.D., Prentice R., Fefer A., Buckner C.D., Storb R. (1979). Antileukemic effect of graft-versus-host disease in human recipients of allogeneic-marrow grafts. N. Engl. J. Med..

[29] Rosenberg S.A., Packard B.S., Aebersold P.M., Solomon D., Topalian S.L., Toy S.T., Simon P., Lotze M.T., Yang J.C., Seipp C.A. (1988). Use of tumor-infiltrating lymphocytes and interleukin-2 in the immunotherapy of patients with metastatic melanoma. A preliminary report. N. Engl. J. Med..

[30] Yee C., Thompson J.A., Byrd D., Riddell S.R., Roche P., Celis E., Greenberg P.D. (2002). Adoptive T cell therapy using antigen-specific CD8+ T cell clones for the treatment of patients with metastatic melanoma: In vivo persistence, migration, and antitumor effect of transferred T cells. Proc. Natl. Acad. Sci. USA.

[31] Sadelain M., Brentjens R., Rivière I. (2009). The promise and potential pitfalls of chimeric antigen receptors. Curr. Opin. Immunol..

[32] Eshhar Z., Bach N., Fitzer-Attas C.J., Gross G., Lustgarten J., Waks T., Schindler D.G. (1996). The T-body approach: Potential for cancer immunotherapy. Springer Semin. Immunopathol..

[33] Kuwana Y., Asakura Y., Utsunomiya N., Nakanishi M., Arata Y., Itoh S., Nagase F., Kurosawa Y. (1987). Expression of chimeric receptor composed of immunoglobulin-derived V regions and T-cell receptor-derived C regions. Biochem. Biophys. Res. Commun..

[34] Becker M.L., Near R., Mudgett-Hunter M., Margolies M.N., Kubo R.T., Kaye J., Hedrick S.M. (1989). Expression of a hybrid immunoglobulin-T cell receptor protein in transgenic mice. Cell.

[35] Gross G., Gorochov G., Waks T., Eshhar Z. (1989). Generation of effector T cells expressing chimeric T cell receptor with antibody type-specificity. Transplant. Proc..

[36] Gross G., Waks T., Eshhar Z. (1989). Expression of immunoglobulin-T-cell receptor chimeric molecules as functional receptors with antibody-type specificity. Proc. Natl. Acad. Sci. USA.

[37] Goverman J., Gomez S.M., Segesman K.D., Hunkapiller T., Laug W.E., Hood L. (1990). Chimeric immunoglobulin-T cell receptor proteins form functional receptors: Implications for T cell receptor complex formation and activation. Cell.

[38] Eshhar Z., Waks T., Gross G., Schindler D.G. (1993). Specific activation and targeting of cytotoxic lymphocytes through chimeric single chains consisting of antibody-binding domains and the gamma or zeta subunits of the immunoglobulin and T-cell receptors. Proc. Natl. Acad. Sci. USA.

[39] Kershaw M.H., Westwood J.A., Parker L.L., Wang G., Eshhar Z., Mavroukakis S.A., White D.E., Wunderlich J.R., Canevari S., Rogers-Freezer L. (2006). A phase I study on adoptive immunotherapy using gene-modified T cells for ovarian cancer. Clin. Cancer Res..

[40] Park J.R., Digiusto D.L., Slovak M., Wright C., Naranjo A., Wagner J., Meechoovet H.B., Bautista C., Chang W.-C., Ostberg J.R. (2007). Adoptive transfer of chimeric antigen receptor re-directed cytolytic T lymphocyte clones in patients with neuroblastoma. Mol. Ther..

[41] Lamers C.H.J., Sleijfer S., Vulto A.G., Kruit W.H.J., Kliffen M., Debets R., Gratama J.W., Stoter G., Oosterwijk E. (2006). Treatment of metastatic renal cell carcinoma with autologous T-lymphocytes genetically retargeted against carbonic anhydrase IX: First clinical experience. J. Clin. Oncol..

[42] Imai C., Mihara K., Andreansky M., Nicholson I.C., Pui C.-H., Geiger T.L., Campana D. (2004). Chimeric receptors with 4-1BB signaling capacity provoke potent cytotoxicity against acute lymphoblastic leukemia. Leukemia.

[43] Maher J., Brentjens R.J., Gunset G., Rivière I., Sadelain M. (2002). Human T-lymphocyte cytotoxicity and proliferation directed by a single chimeric TCRzeta /CD28 receptor. Nat. Biotechnol..

[44] ClinicalTrials.gov NCT02435849—Study of Efficacy and Safety of CTL019 in Pediatric ALL Patients (ELIANA). NCT02435849.

[45] ClinicalTrials.gov NCT02445248—Study of Efficacy and Safety of CTL019 in Adult DLBCL Patients (JULIET). NCT02445248.

[46] ClinicalTrials.gov NCT02228096—Study of Efficacy and Safety of CTL019 in Pediatric ALL Patients (ENSIGN). NCT02228096.

[47] ClinicalTrials.gov NCT03568461—Efficacy and Safety of Tisagenlecleucel in Adult Patients with Refractory or Relapsed Follicular Lymphoma (ELARA). NCT03568461.

[48] ClinicalTrials.gov NCT01029366—CART19 to Treat B-Cell Leukemia or Lymphoma That Are Resistant or Refractory to Chemotherapy. NCT01029366.

[49] ClinicalTrials.gov NCT01747486—Dose Optimization Trial of CD19 Redirected Autologous T Cells. NCT01747486.

[50] ClinicalTrials.gov NCT02030847—Study of Redirected Autologous T Cells Engineered to Contain Anti-CD19 Attached to TCR and 4-1BB Signaling Domains in Patients with Chemotherapy Resistant or Refractory Acute Lymphoblastic Leukemia. NCT02030847.

[51] ClinicalTrials.gov NCT02030834—Phase IIa Study of Redirected Autologous T Cells Engineered to Contain Anti-CD19 Attached to TCRz and 4-Signaling Domains in Patients with Chemotherapy Relapsed or Refractory CD19+ Lymphomas. NCT02030834.

[52] Schuster S.J., Bishop M.R., Tam C.S., Waller E.K., Borchmann P., McGuirk J.P., Jäger U., Jaglowski S., Andreadis C., Westin J.R. (2019). Tisagenlecleucel in Adult Relapsed or Refractory Diffuse Large B-Cell Lymphoma. N. Engl. J. Med..

[53] ClinicalTrials.gov NCT02348216—Study Evaluating the Safety and Efficacy of KTE-C19 in Adult Participants with Refractory Aggressive Non-Hodgkin Lymphoma (ZUMA-1). NCT02348216.

[54] ClinicalTrials.gov NCT03105336—A Phase 2 Multicenter Study of Axicabtagene Ciloleucel in Subjects with Relapsed/Refractory Indolent Non-Hodgkin Lymphoma (ZUMA-5). NCT03105336.

[55] ClinicalTrials.gov NCT03153462—Axicabtagene Ciloleucel Expanded Access Study (ZUMA-9). NCT03153462.

[56] ClinicalTrials.gov NCT03761056—Study to Evaluate the Efficacy and Safety of Axicabtagene Ciloleucel as First-Line Therapy in Participants with High-Risk Large B-Cell Lymphoma (ZUMA-12). NCT03761056.

[57] ClinicalTrials.gov NCT03391466—Study of Effectiveness of Axicabtagene Ciloleucel Compared to Standard of Care Therapy in Patients with Relapsed/Refractory Diffuse Large B Cell Lymphoma (ZUMA-7). NCT03391466.

[58] Sadelain M., Rivière I., Brentjens R. (2003). Targeting tumours with genetically enhanced T lymphocytes. Nat. Rev. Cancer.

[59] Mezzanzanica D., Canevari S., Mazzoni A., Figini M., Colnaghi M.I., Waks T., Schindler D.G., Eshhar Z. (1998). Transfer of chimeric receptor gene made of variable regions of tumor-specific antibody confers anticarbohydrate specificity on T cells. Cancer Gene Ther..

[60] Krause A., Guo H.F., Latouche J.B., Tan C., Cheung N.K., Sadelain M. (1998). Antigen-dependent CD28 signaling selectively enhances survival and proliferation in genetically modified activated human primary T lymphocytes. J. Exp. Med..

[61] Altenschmidt U., Klundt E., Groner B. (1997). Adoptive transfer of in vitro-targeted, activated T lymphocytes results in total tumor regression. J. Immunol..

[62] Darcy P.K., Haynes N.M., Snook M.B., Trapani J.A., Cerruti L., Jane S.M., Smyth M.J. (2000). Redirected perforin-dependent lysis of colon carcinoma by ex vivo genetically engineered CTL. J. Immunol..

[63] Haynes N.M., Trapani J.A., Teng M.W.L., Jackson J.T., Cerruti L., Jane S.M., Kershaw M.H., Smyth M.J., Darcy P.K. (2002). Single-chain antigen recognition receptors that costimulate potent rejection of established experimental tumors. Blood.

[64] McGuinness R.P., Ge Y., Patel S.D., Kashmiri S.V., Lee H.S., Hand P.H., Schlom J., Finer M.H., McArthur J.G. (1999). Anti-tumor activity of human T cells expressing the CC49-zeta chimeric immune receptor. Hum. Gene Ther..

[65] Hwu P., Yang J.C., Cowherd R., Treisman J., Shafer G.E., Eshhar Z., Rosenberg S.A. (1995). In vivo antitumor activity of T cells redirected with chimeric antibody/T-cell receptor genes. Cancer Res..

[66] Wang G., Chopra R.K., Royal R.E., Yang J.C., Rosenberg S.A., Hwu P. (1998). A T cell-independent antitumor response in mice with bone marrow cells retrovirally transduced with an antibody/Fc-gamma chain chimeric receptor gene recognizing a human ovarian cancer antigen. Nat. Med..

[67] Jenkins M.K., Taylor P.S., Norton S.D., Urdahl K.B. (1991). CD28 delivers a costimulatory signal involved in antigen-specific IL-2 production by human T cells. J. Immunol..

[68] Staveley-O’Carroll K., Sotomayor E., Montgomery J., Borrello I., Hwang L., Fein S., Pardoll D., Levitsky H. (1998). Induction of antigen-specific T cell anergy: An early event in the course of tumor progression. Proc. Natl. Acad. Sci. USA.

[69] Hombach A., Sent D., Schneider C., Heuser C., Koch D., Pohl C., Seliger B., Abken H. (2001). T-cell activation by recombinant receptors: CD28 costimulation is required for interleukin 2 secretion and receptor-mediated T-cell proliferation but does not affect receptor-mediated target cell lysis. Cancer Res..

[70] Liebowitz D.N., Lee K.P., June C.H. (1998). Costimulatory approaches to adoptive immunotherapy. Curr. Opin. Oncol..

[71] June C.H. (2007). Adoptive T cell therapy for cancer in the clinic. J. Clin. Investig..

[72] Friedmann-Morvinski D., Bendavid A., Waks T., Schindler D., Eshhar Z. (2005). Redirected primary T cells harboring a chimeric receptor require costimulation for their antigen-specific activation. Blood.

[73] Finney H.M., Lawson A.D., Bebbington C.R., Weir A.N. (1998). Chimeric receptors providing both primary and costimulatory signaling in T cells from a single gene product. J. Immunol..

[74] Milone M.C., Fish J.D., Carpenito C., Carroll R.G., Binder G.K., Teachey D., Samanta M., Lakhal M., Gloss B., Danet-Desnoyers G. (2009). Chimeric receptors containing CD137 signal transduction domains mediate enhanced survival of T cells and increased antileukemic efficacy in vivo. Mol. Ther..

[75] Carpenito C., Milone M.C., Hassan R., Simonet J.C., Lakhal M., Suhoski M.M., Varela-Rohena A., Haines K.M., Heitjan D.F., Albelda S.M. (2009). Control of large, established tumor xenografts with genetically retargeted human T cells containing CD28 and CD137 domains. Proc. Natl. Acad. Sci. USA.

[76] Young R.M., Engel N.W., Uslu U., Wellhausen N., June C.H. (2022). Next-Generation CAR T-cell Therapies. Cancer Discov..

[77] Baker D.J., Arany Z., Baur J.A., Epstein J.A., June C.H. (2023). CAR T therapy beyond cancer: The evolution of a living drug. Nature.

[78] Sadelain M., Rivière I., Riddell S. (2017). Therapeutic T cell engineering. Nature.

[79] Labanieh L., Mackall C.L. (2023). CAR immune cells: Design principles, resistance and the next generation. Nature.

[80] Singh N., Maus M.V. (2023). Synthetic manipulation of the cancer-immunity cycle: CAR-T cell therapy. Immunity.

[81] Neelapu S.S., Chavez J.C., Sehgal A., Epperla N., Ulrickson M.L., Bachy E., Munshi P., Casulo C., Maloney D.G., Vos S.d. (2023). Three-Year Follow-up Analysis of Axicabtagene Ciloleucel in Relapsed/Refractory Indolent Non-Hodgkin Lymphoma (ZUMA-5). Blood.

[82] Locke F.L., Miklos D.B., Jacobson C.A., Perales M.-A., Kersten M.-J., Oluwole O.O., Ghobadi A., Rapoport A.P., McGuirk J., Pagel J.M. (2022). Axicabtagene Ciloleucel as Second-Line Therapy for Large B-Cell Lymphoma. N. Engl. J. Med..

[83] ClinicalTrials.gov NCT02601313—Study of Brexucabtagene Autoleucel (KTE-X19) in Participants with Relapsed/Refractory Mantle Cell Lymphoma (Cohort 1 and Cohort 2) (ZUMA-2). NCT02601313.

[84] ClinicalTrials.gov NCT02614066—A Study Evaluating the Safety and Efficacy of Brexucabtagene Autoleucel (KTE-X19) in Adult Subjects with Relapsed/Refractory B-precursor Acute Lymphoblastic Leukemia (ZUMA-3). NCT02614066.

[85] ClinicalTrials.gov NCT02625480—Study Evaluating Brexucabtagene Autoleucel (KTE-X19) in Pediatric and Adolescent Participants with Relapsed/Refractory B-precursor Acute Lymphoblastic Leukemia or Relapsed/Refractory B-Cell Non-Hodgkin Lymphoma (ZUMA-4). NCT02625480.

[86] ClinicalTrials.gov NCT03624036—Study to Evaluate the Safety and Tolerability of Brexucabtagene Autoleucel (KTE-X19) in People with Relapsed/Refractory Chronic Lymphocytic Leukemia and Small Lymphocytic Lymphoma (ZUMA-8). NCT03624036.

[87] Goy A., Jacobson C.A., Flinn I.W., Hill B.T., Weng W.K., Mountjoy L., Olalekan O., Zheng D., Nunes A., Zhang W. (2023). Outcomes of Patients with Relapsed/Refractory Mantle Cell Lymphoma (R/R MCL) Treated with Brexucabtagene Autoleucel (Brexu-cel) in ZUMA-2 and ZUMA-18, an Expanded Access Study. Blood.

[88] Wang M., Munoz J., Goy A., Locke F.L., Jacobson C.A., Hill B.T., Timmerman J.M., Holmes H., Jaglowski S., Flinn I.W. (2020). KTE-X19 CAR T-Cell Therapy in Relapsed or Refractory Mantle-Cell Lymphoma. N. Engl. J. Med..

[89] Shah B.D., Bishop M.R., Oluwole O.O., Logan A.C., Baer M.R., Donnellan W.B., O’Dwyer K.M., Holmes H., Arellano M.L., Ghobadi A. (2021). KTE-X19 anti-CD19 CAR T-cell therapy in adult relapsed/refractory acute lymphoblastic leukemia: ZUMA-3 phase 1 results. Blood.

[90] Shah B.D., Ghobadi A., Oluwole O.O., Logan A.C., Boissel N., Cassaday R.D., Leguay T., Bishop M.R., Topp M.S., Tzachanis D. (2021). KTE-X19 for relapsed or refractory adult B-cell acute lymphoblastic leukaemia: Phase 2 results of the single-arm, open-label, multicentre ZUMA-3 study. Lancet.

[91] ClinicalTrials.gov NCT03361748—Efficacy and Safety Study of bb2121 in Subjects with Relapsed and Refractory Multiple Myeloma (KarMMa). NCT03361748.

[92] ClinicalTrials.gov NCT03601078—An Efficacy and Safety Study of bb2121 in Subjects with Relapsed and Refractory Multiple Myeloma and in Subjects with High-Risk Multiple Myeloma (KarMMa-2). NCT03601078.

[93] ClinicalTrials.gov NCT03651128—Efficacy and Safety Study of bb2121 Versus Standard Regimens in Subjects with Relapsed and Refractory Multiple Myeloma (RRMM) (KarMMa-3). NCT03651128.

[94] ClinicalTrials.gov NCT02658929—Study of bb2121 in Multiple Myeloma. NCT02658929.

[95] ClinicalTrials.gov NCT02786511—Longterm Follow-up of Subjects Treated with bb2121. NCT02786511.

[96] Munshi N.C., Anderson L.D., Shah N., Madduri D., Berdeja J., Lonial S., Raje N., Lin Y., Siegel D., Oriol A. (2021). Idecabtagene Vicleucel in Relapsed and Refractory Multiple Myeloma. N. Engl. J. Med..

[97] Rodriguez-Otero P., Ailawadhi S., Arnulf B., Patel K., Cavo M., Nooka A.K., Manier S., Callander N., Costa L.J., Vij R. (2023). Ide-cel or Standard Regimens in Relapsed and Refractory Multiple Myeloma. N. Engl. J. Med..

[98] ClinicalTrials.gov NCT02631044—Study Evaluating the Safety and Pharmacokinetics of JCAR017 in B-cell Non-Hodgkin Lymphoma (TRANSCEND-NHL-001). NCT02631044.

[99] ClinicalTrials.gov NCT03484702—Trial to Determine the Efficacy and Safety of JCAR017 in Adult Participants with Aggressive B-Cell Non-Hodgkin Lymphoma (TRANSCENDWORLD). NCT03484702.

[100] ClinicalTrials.gov NCT03744676—A Safety Trial of Lisocabtagene Maraleucel (JCAR017) for Relapsed and Refractory (R/R) B-cell Non-Hodgkin Lymphoma (NHL) in the Outpatient Setting (TRANSCEND-OUTREACH-007). NCT03744676.

[101] ClinicalTrials.gov NCT03310619—A Safety and Efficacy Trial of JCAR017 Combinations in Subjects with Relapsed/Refractory B-cell Malignancies (PLATFORM). NCT03310619.

[102] ClinicalTrials.gov NCT03483103—Lisocabtagene Maraleucel (JCAR017) as Second-Line Therapy (TRANSCEND-PILOT-017006). NCT03483103.

[103] ClinicalTrials.gov NCT03331198—Study Evaluating Safety and Efficacy of JCAR017 in Subjects with Relapsed or Refractory Chronic Lymphocytic Leukemia (CLL) or Small Lymphocytic Lymphoma (SLL). NCT03331198.

[104] ClinicalTrials.gov NCT03743246—A Study to Evaluate the Safety and Efficacy of JCAR017 in Pediatric Subjects with Relapsed/Refractory (r/r) B-cell Acute Lymphoblastic Leukemia (B-ALL) and B-cell Non-Hodgkin Lymphoma (B-NHL). NCT03743246.

[105] ClinicalTrials.gov NCT03575351—A Study to Compare the Efficacy and Safety of JCAR017 to Standard of Care in Adult Subjects with High-risk, Transplant-eligible Relapsed or Refractory Aggressive B-cell Non-Hodgkin Lymphomas (TRANSFORM). NCT03575351.

[106] Abramson J.S., Palomba M.L., Gordon L.I., Lunning M.A., Wang M., Arnason J., Mehta A., Purev E., Maloney D.G., Andreadis C. (2020). Lisocabtagene maraleucel for patients with relapsed or refractory large B-cell lymphomas (TRANSCEND NHL 001): A multicentre seamless design study. Lancet.

[107] Abramson J.S., Palomba M.L., Gordon L.I., Lunning M., Wang M., Arnason J., Purev E., Maloney D.G., Andreadis C., Sehgal A. (2024). Two-year follow-up of lisocabtagene maraleucel in relapsed or refractory large B-cell lymphoma in TRANSCEND NHL 001. Blood.

[108] Sehgal A., Hoda D., Riedell P.A., Ghosh N., Hamadani M., Hildebrandt G.C., Godwin J.E., Reagan P.M., Wagner-Johnston N., Essell J. (2022). Lisocabtagene maraleucel as second-line therapy in adults with relapsed or refractory large B-cell lymphoma who were not intended for haematopoietic stem cell transplantation (PILOT): An open-label, phase 2 study. Lancet Oncol..

[109] Abramson J.S., Solomon S.R., Arnason J., Johnston P.B., Glass B., Bachanova V., Ibrahimi S., Mielke S., Mutsaers P., Hernandez-Ilizaliturri F. (2023). Lisocabtagene maraleucel as second-line therapy for large B-cell lymphoma: Primary analysis of the phase 3 TRANSFORM study. Blood.

[110] ClinicalTrials.gov NCT03548207—A Study of JNJ-68284528, a Chimeric Antigen Receptor T Cell (CAR-T) Therapy Directed against B-Cell Maturation Antigen (BCMA) in Participants with Relapsed or Refractory Multiple Myeloma (CARTITUDE-1). NCT03548207.

[111] ClinicalTrials.gov NCT04133636—A Study of JNJ-68284528, a Chimeric Antigen Receptor T Cell (CAR-T) Therapy Directed against B-cell Maturation Antigen (BCMA) in Participants with Multiple Myeloma (CARTITUDE-2). NCT04133636.

[112] ClinicalTrials.gov NCT04181827—A Study Comparing JNJ-68284528, a CAR-T Therapy Directed against B-cell Maturation Antigen (BCMA), Versus Pomalidomide, Bortezomib and Dexamethasone (PVd) or Daratumumab, Pomalidomide and Dexamethasone (DPd) in Participants with Relapsed and Lenalidomide-Refractory Multiple Myeloma (CARTITUDE-4). NCT04181827.

[113] Berdeja J.G., Madduri D., Usmani S.Z., Jakubowiak A., Agha M., Cohen A.D., Stewart A.K., Hari P., Htut M., Lesokhin A. (2021). Ciltacabtagene autoleucel, a B-cell maturation antigen-directed chimeric antigen receptor T-cell therapy in patients with relapsed or refractory multiple myeloma (CARTITUDE-1): A phase 1b/2 open-label study. Lancet.

[114] Martin T., Usmani S.Z., Berdeja J.G., Agha M., Cohen A.D., Hari P., Avigan D., Deol A., Htut M., Lesokhin A. (2023). Ciltacabtagene Autoleucel, an Anti-B-cell Maturation Antigen Chimeric Antigen Receptor T-Cell Therapy, for Relapsed/Refractory Multiple Myeloma: CARTITUDE-1 2-Year Follow-up. J. Clin. Oncol..

[115] Cohen A.D., Mateos M.-V., Cohen Y.C., Rodriguez-Otero P., Paiva B., van de Donk N.W.C.J., Martin T., Suvannasankha A., de Braganca K.C., Corsale C. (2023). Efficacy and safety of cilta-cel in patients with progressive multiple myeloma after exposure to other BCMA-targeting agents. Blood.

[116] Porter D.L., Levine B.L., Kalos M., Bagg A., June C.H. (2011). Chimeric antigen receptor-modified T cells in chronic lymphoid leukemia. N. Engl. J. Med..

[117] Kalos M., Levine B.L., Porter D.L., Katz S., Grupp S.A., Bagg A., June C.H. (2011). T cells with chimeric antigen receptors have potent antitumor effects and can establish memory in patients with advanced leukemia. Sci. Transl. Med..

[118] Grupp S.A., Kalos M., Barrett D., Aplenc R., Porter D.L., Rheingold S.R., Teachey D.T., Chew A., Hauck B., Wright J.F. (2013). Chimeric antigen receptor-modified T cells for acute lymphoid leukemia. N. Engl. J. Med..

[119] ClinicalTrials.gov NCT01626495—Phase I/IIA Study of CART19 Cells for Patients with Chemotherapy Resistant or Refractory CD19+ Leukemia and Lymphoma (Pedi CART19). NCT01626495.

[120] Maude S.L., Frey N., Shaw P.A., Aplenc R., Barrett D.M., Bunin N.J., Chew A., Gonzalez V.E., Zheng Z., Lacey S.F. (2014). Chimeric antigen receptor T cells for sustained remissions in leukemia. N. Engl. J. Med..

[121] Maude S.L., Laetsch T.W., Buechner J., Rives S., Boyer M., Bittencourt H., Bader P., Verneris M.R., Stefanski H.E., Myers G.D. (2018). Tisagenlecleucel in Children and Young Adults with B-Cell Lymphoblastic Leukemia. N. Engl. J. Med..

[122] Maude S.L., Pulsipher M.A., Boyer M.W., Grupp S.A., Davies S.M., Phillips C.L., Verneris M.R., August K.J., Schlis K., Driscoll T.A. (2016). Efficacy and Safety of CTL019 in the First US Phase II Multicenter Trial in Pediatric Relapsed/Refractory Acute Lymphoblastic Leukemia: Results of an Interim Analysis. Blood.

[123] Laetsch T.W., Maude S.L., Rives S., Hiramatsu H., Bittencourt H., Bader P., Baruchel A., Boyer M., de Moerloose B., Qayed M. (2023). Three-Year Update of Tisagenlecleucel in Pediatric and Young Adult Patients with Relapsed/Refractory Acute Lymphoblastic Leukemia in the ELIANA Trial. J. Clin. Oncol..

[124] Fowler N.H., Dickinson M., Dreyling M., Martinez-Lopez J., Kolstad A., Butler J., Ghosh M., Popplewell L., Chavez J.C., Bachy E. (2022). Tisagenlecleucel in adult relapsed or refractory follicular lymphoma: The phase 2 ELARA trial. Nat. Med..

[125] O’Leary M.C., Lu X., Huang Y., Lin X., Mahmood I., Przepiorka D., Gavin D., Lee S., Liu K., George B. (2019). FDA Approval Summary: Tisagenlecleucel for Treatment of Patients with Relapsed or Refractory B-cell Precursor Acute Lymphoblastic Leukemia. Clin. Cancer Res..

[126] Salles G., Schuster S.J., Dreyling M., Fischer L., Kuruvilla J., Patten P.E.M., von Tresckow B., Smith S.M., Jiménez-Ubieto A., Davis K.L. (2022). Efficacy comparison of tisagenlecleucel vs. usual care in patients with relapsed or refractory follicular lymphoma. Blood Adv..

[127] Kochenderfer J.N., Feldman S.A., Zhao Y., Xu H., Black M.A., Morgan R.A., Wilson W.H., Rosenberg S.A. (2009). Construction and preclinical evaluation of an anti-CD19 chimeric antigen receptor. J. Immunother..

[128] Nicholson I.C., Lenton K.A., Little D.J., Decorso T., Lee F.T., Scott A.M., Zola H., Hohmann A.W. (1997). Construction and characterisation of a functional CD19 specific single chain Fv fragment for immunotherapy of B lineage leukaemia and lymphoma. Mol. Immunol..

[129] Kochenderfer J.N., Dudley M.E., Kassim S.H., Somerville R.P.T., Carpenter R.O., Stetler-Stevenson M., Yang J.C., Phan G.Q., Hughes M.S., Sherry R.M. (2015). Chemotherapy-refractory diffuse large B-cell lymphoma and indolent B-cell malignancies can be effectively treated with autologous T cells expressing an anti-CD19 chimeric antigen receptor. J. Clin. Oncol..

[130] Kochenderfer J.N., Somerville R.P.T., Lu T., Yang J.C., Sherry R.M., Feldman S.A., McIntyre L., Bot A., Rossi J., Lam N. (2017). Long-Duration Complete Remissions of Diffuse Large B Cell Lymphoma after Anti-CD19 Chimeric Antigen Receptor T Cell Therapy. Mol. Ther..

[131] ClinicalTrials.gov NCT00924326—CAR T Cell Receptor Immunotherapy for Patients with B-Cell Lymphoma. NCT00924326.

[132] Locke F.L., Neelapu S.S., Bartlett N.L., Siddiqi T., Chavez J.C., Hosing C.M., Ghobadi A., Budde L.E., Bot A., Rossi J.M. (2017). Phase 1 Results of ZUMA-1: A Multicenter Study of KTE-C19 Anti-CD19 CAR T Cell Therapy in Refractory Aggressive Lymphoma. Mol. Ther..

[133] Neelapu S.S., Locke F.L., Bartlett N.L., Lekakis L.J., Miklos D.B., Jacobson C.A., Braunschweig I., Oluwole O.O., Siddiqi T., Lin Y. (2017). Axicabtagene Ciloleucel CAR T-Cell Therapy in Refractory Large B-Cell Lymphoma. N. Engl. J. Med..

[134] Neelapu S.S., Jacobson C.A., Ghobadi A., Miklos D.B., Lekakis L.J., Oluwole O.O., Lin Y., Braunschweig I., Hill B.T., Timmerman J.M. (2023). Five-year follow-up of ZUMA-1 supports the curative potential of Axicabtagene ciloleucel in refractory large B-cell lymphoma. Blood.

[135] Westin J.R., Oluwole O.O., Kersten M.J., Miklos D.B., Perales M.-A., Ghobadi A., Rapoport A.P., Sureda A., Jacobson C.A., Farooq U. (2023). Survival with Axicabtagene ciloleucel in Large B-Cell Lymphoma. N. Engl. J. Med..

[136] Jacobson C.A., Chavez J.C., Sehgal A.R., William B.M., Munoz J., Salles G., Munshi P.N., Casulo C., Maloney D.G., Vos S.d. (2022). Axicabtagene ciloleucel in relapsed or refractory indolent non-Hodgkin lymphoma (ZUMA-5): A single-arm, multicentre, phase 2 trial. Lancet Oncol..

[137] Raje N., Berdeja J., Lin Y., Siegel D., Jagannath S., Madduri D., Liedtke M., Rosenblatt J., Maus M.V., Turka A. (2019). Anti-BCMA CAR T-Cell Therapy bb2121 in Relapsed or Refractory Multiple Myeloma. N. Engl. J. Med..

[138] Friedman K.M., Garrett T.E., Evans J.W., Horton H.M., Latimer H.J., Seidel S.L., Horvath C.J., Morgan R.A. (2018). Effective Targeting of Multiple B-Cell Maturation Antigen-Expressing Hematological Malignances by Anti-B-Cell Maturation Antigen Chimeric Antigen Receptor T Cells. Hum. Gene Ther..

[139] Tai Y.-T., Anderson K.C. (2015). Targeting B-cell maturation antigen in multiple myeloma. Immunotherapy.

[140] Garfall A.L., Maus M.V., Hwang W.-T., Lacey S.F., Mahnke Y.D., Melenhorst J.J., Zheng Z., Vogl D.T., Cohen A.D., Weiss B.M. (2015). Chimeric Antigen Receptor T Cells against CD19 for Multiple Myeloma. N. Engl. J. Med..

[141] Ali S.A., Shi V., Maric I., Wang M., Stroncek D.F., Rose J.J., Brudno J.N., Stetler-Stevenson M., Feldman S.A., Hansen B.G. (2016). T cells expressing an anti-B-cell maturation antigen chimeric antigen receptor cause remissions of multiple myeloma. Blood.

[142] Sommermeyer D., Hudecek M., Kosasih P.L., Gogishvili T., Maloney D.G., Turtle C.J., Riddell S.R. (2016). Chimeric antigen receptor-modified T cells derived from defined CD8+ and CD4+ subsets confer superior antitumor reactivity in vivo. Leukemia.

[143] Teoh J., Brown L.F. (2022). Developing lisocabtagene maraleucel chimeric antigen receptor T-cell manufacturing for improved process, product quality and consistency across CD19+ hematologic indications. Cytotherapy.

[144] Martin T., Lin Y., Agha M., Cohen A.D., Htut M., Stewart A.K., Hari P., Berdeja J.G., Usmani S.Z., Yeh T.-M. (2022). Health-related quality of life in patients given ciltacabtagene autoleucel for relapsed or refractory multiple myeloma (CARTITUDE-1): A phase 1b-2, open-label study. Lancet Haematol..

[145] Zhao W.-H., Liu J., Wang B.-Y., Chen Y.-X., Cao X.-M., Yang Y., Zhang Y.-L., Wang F.-X., Zhang P.-Y., Lei B. (2018). A phase 1, open-label study of LCAR-B38M, a chimeric antigen receptor T cell therapy directed against B cell maturation antigen, in patients with relapsed or refractory multiple myeloma. J. Hematol. Oncol..

[146] San-Miguel J., Dhakal B., Yong K., Spencer A., Anguille S., Mateos M.-V., Fernández de Larrea C., Martínez-López J., Moreau P., Touzeau C. (2023). Cilta-cel or Standard Care in Lenalidomide-Refractory Multiple Myeloma. N. Engl. J. Med..

[147] Cappell K.M., Kochenderfer J.N. (2023). Long-term outcomes following CAR T cell therapy: What we know so far. Nat. Rev. Clin. Oncol..

[148] Lee D.W., Santomasso B.D., Locke F.L., Ghobadi A., Turtle C.J., Brudno J.N., Maus M.V., Park J.H., Mead E., Pavletic S. (2019). ASTCT Consensus Grading for Cytokine Release Syndrome and Neurologic Toxicity Associated with Immune Effector Cells. Biol. Blood Marrow Transplant..

[149] Rejeski K., Subklewe M., Aljurf M., Bachy E., Balduzzi A., Barba P., Bruno B., Benjamin R., Carrabba M.G., Chabannon C. (2023). Immune effector cell-associated hematotoxicity: EHA/EBMT consensus grading and best practice recommendations. Blood.

[150] Hayden P.J., Roddie C., Bader P., Basak G.W., Bonig H., Bonini C., Chabannon C., Ciceri F., Corbacioglu S., Ellard R. (2022). Management of adults and children receiving CAR T-cell therapy: 2021 best practice recommendations of the European Society for Blood and Marrow Transplantation (EBMT) and the Joint Accreditation Committee of ISCT and EBMT (JACIE) and the European Haematology Association (EHA). Ann. Oncol..

[151] Maus M.V., Alexander S., Bishop M.R., Brudno J.N., Callahan C., Davila M.L., Diamonte C., Dietrich J., Fitzgerald J.C., Frigault M.J. (2020). Society for Immunotherapy of Cancer (SITC) clinical practice guideline on immune effector cell-related adverse events. J. Immunother. Cancer.

[152] Fried S., Avigdor A., Bielorai B., Meir A., Besser M.J., Schachter J., Shimoni A., Nagler A., Toren A., Jacoby E. (2019). Early and late hematologic toxicity following CD19 CAR-T cells. Bone Marrow Transplant..

[153] Rejeski K., Perez A., Sesques P., Hoster E., Berger C., Jentzsch L., Mougiakakos D., Frölich L., Ackermann J., Bücklein V. (2021). CAR-HEMATOTOX: A model for CAR T-cell-related hematologic toxicity in relapsed/refractory large B-cell lymphoma. Blood.

[154] Rejeski K., Greco R., Onida F., Sánchez-Ortega I., Bonini C., Sureda A., Gribben J.G., Yakoub-Agha I., Subklewe M. (2023). An International Survey on Grading, Diagnosis, and Management of Immune Effector Cell-Associated Hematotoxicity (ICAHT) following CAR T-cell Therapy on Behalf of the EBMT and EHA. Hemasphere.

[155] Juluri K.R., Wu Q.V., Voutsinas J., Hou J., Hirayama A.V., Mullane E., Miles N., Maloney D.G., Turtle C.J., Bar M. (2022). Severe cytokine release syndrome is associated with hematologic toxicity following CD19 CAR T-cell therapy. Blood Adv..

[156] Jain T., Knezevic A., Pennisi M., Chen Y., Ruiz J.D., Purdon T.J., Devlin S.M., Smith M., Shah G.L., Halton E. (2020). Hematopoietic recovery in patients receiving chimeric antigen receptor T-cell therapy for hematologic malignancies. Blood Adv..

[157] Sandler R.D., Tattersall R.S., Schoemans H., Greco R., Badoglio M., Labopin M., Alexander T., Kirgizov K., Rovira M., Saif M. (2020). Diagnosis and Management of Secondary HLH/MAS following HSCT and CAR-T Cell Therapy in Adults; A Review of the Literature and a Survey of Practice within EBMT Centres on Behalf of the Autoimmune Diseases Working Party (ADWP) and Transplant Complications Working Party (TCWP). Front. Immunol..

[158] Neelapu S.S., Tummala S., Kebriaei P., Wierda W., Gutierrez C., Locke F.L., Komanduri K.V., Lin Y., Jain N., Daver N. (2018). Chimeric antigen receptor T-cell therapy—Assessment and management of toxicities. Nat. Rev. Clin. Oncol..

[159] Hines M.R., Knight T.E., McNerney K.O., Leick M.B., Jain T., Ahmed S., Frigault M.J., Hill J.A., Jain M.D., Johnson W.T. (2023). Immune Effector Cell-Associated Hemophagocytic Lymphohistiocytosis-Like Syndrome. Transplant. Cell. Ther..

[160] Mullanfiroze K., Lazareva A., Chu J., Williams L., Burridge S., Silva J., Chiesa R., Rao K., Lucchini G., Ghorashian S. (2022). CD34+-selected stem cell boost can safely improve cytopenias following CAR T-cell therapy. Blood Adv..

[161] Gagelmann N., Wulf G.G., Duell J., Glass B., van Heteren P., von Tresckow B., Fischer M., Penack O., Ayuk F., Einsele H. (2023). Hematopoietic stem cell boost for persistent neutropenia after CAR T-cell therapy: A GLA/DRST study. Blood Adv..

[162] Shimabukuro-Vornhagen A., Gödel P., Subklewe M., Stemmler H.J., Schlößer H.A., Schlaak M., Kochanek M., Böll B., von Bergwelt-Baildon M.S. (2018). Cytokine release syndrome. J. Immunother. Cancer.

[163] National Cancer Institute Common Terminology Criteria for Adverse Events (CTCAE). https://ctep.cancer.gov/protocoldevelopment/electronic_applications/ctc.htm.

[164] Lee D.W., Gardner R., Porter D.L., Louis C.U., Ahmed N., Jensen M., Grupp S.A., Mackall C.L. (2014). Current concepts in the diagnosis and management of cytokine release syndrome. Blood.

[165] Park J.H., Rivière I., Gonen M., Wang X., Sénéchal B., Curran K.J., Sauter C., Wang Y., Santomasso B., Mead E. (2018). Long-Term Follow-up of CD19 CAR Therapy in Acute Lymphoblastic Leukemia. N. Engl. J. Med..

[166] Majumder A. (2023). Evolving CAR-T-Cell Therapy for Cancer Treatment: From Scientific Discovery to Cures. Cancers.

[167] Santomasso B.D., Gust J., Perna F. (2023). How I treat unique and difficult-to-manage cases of CAR T-cell therapy-associated neurotoxicity. Blood.

[168] Nguyen D.T., Ogando-Rivas E., Liu R., Wang T., Rubin J., Jin L., Tao H., Sawyer W.W., Mendez-Gomez H.R., Cascio M. (2022). CAR T Cell Locomotion in Solid Tumor Microenvironment. Cells.

[169] Bellone M., Calcinotto A. (2013). Ways to enhance lymphocyte trafficking into tumors and fitness of tumor infiltrating lymphocytes. Front. Oncol..

[170] Mahdi J., Dietrich J., Straathof K., Roddie C., Scott B.J., Davidson T.B., Prolo L.M., Batchelor T.T., Campen C.J., Davis K.L. (2023). Tumor inflammation-associated neurotoxicity. Nat. Med..

[171] Ghilardi G., Fraietta J.A., Gerson J.N., van Deerlin V.M., Morrissette J.J.D., Caponetti G.C., Paruzzo L., Harris J.C., Chong E.A., Susanibar Adaniya S.P. (2024). T-cell Lymphoma and Secondary Primary Malignancy Risk after Commercial CAR T-cell Therapy. Nat. Med..

